# Medicinal plants used by the Tamang community in the Makawanpur district of central Nepal

**DOI:** 10.1186/1746-4269-10-5

**Published:** 2014-01-10

**Authors:** Dol Raj Luitel, Maan B Rokaya, Binu Timsina, Zuzana Münzbergová

**Affiliations:** 1 Department of Plant Resources Office, Ministry of Forest and Soil Conservation, Thapathali, Kathmandu, Nepal; 2 Institute of Botany, Academy of Sciences of the Czech Republic, Zamek 1, Průhonice 25243, Czech Republic; 3 Department of Biodiversity Research, Global Change Research Centre AS ČR, Na sádkách 7, České Budějovice 37005, Czech Republic; 4 Institute for Environmental Studies/Department of Botany, Faculty of Science, Charles University, Benatska 2, Prague 12801, Czech Republic

**Keywords:** Makawanpur district, Medicinal plants, Pharmacology, Phytochemistry, Tamang community

## Abstract

**Background:**

We can conserve cultural heritage and gain extensive knowledge of plant species with pharmacological potential to cure simple to life-threatening diseases by studying the use of plants in indigenous communities. Therefore, it is important to conduct ethnobotanical studies in indigenous communities and to validate the reported uses of plants by comparing ethnobotanical studies with phytochemical and pharmacological studies.

**Materials and methods:**

This study was conducted in a Tamang community dwelling in the Makawanpur district of central Nepal. We used semi-structured and structured questionnaires during interviews to collect information. We compared use reports with available phytochemical and pharmacological studies for validation.

**Results:**

A total of 161 plant species belonging to 86 families and 144 genera to cure 89 human ailments were documented. Although 68 plant species were cited as medicinal in previous studies, 55 different uses described by the Tamang people were not found in any of the compared studies. Traditional uses for 60 plant species were consistent with pharmacological and phytochemical studies.

**Conclusions:**

The Tamang people in Makawanpur are rich in ethnopharmacological understanding. The present study highlights important medicinal plant species by validating their traditional uses. Different plant species can improve local economies through proper harvesting, adequate management and development of modern techniques to maximize their use.

## Background

Plants have been used for human benefit from time immemorial [1]. In the developing world, 70–80% of the population relies on plants for primary health care [2]. The use of plants as medicine is slowly increasing in the developed world [3] because they have minor or no side effects [4]. Although there is wide use of herbal medicine, traditional knowledge of the use of medicinal plants is influenced by rapid urbanization, migration, climate change, and the increasing number of modern healthcare systems throughout the world, including in Nepal [5-10]. The traditional use of plants by indigenous communities reflects the cultural aspects as well as biodynamic elements that have immense pharmacological potential to cure many diseases [11,12]. The documentation of traditional knowledge aids in the preservation of indigenous culture, identifies threatened species and contributes to the conservation and management of plant diversity [13,14]. The cultural and biological diversity of Nepal offers immense opportunities for ethnobotanical studies [7,15-17]. In Nepali traditional medicine, more than 2300 plant species [18] are used by 125 caste/ethnic communities speaking approximately 123 different languages [19].

In addition to documenting the traditional knowledge related to medicinal plants, scientific validation of traditional medicinal plants has been an important path of recent research [20]. Validation is performed by *in-vitro*[21-23] or *in-vivo* experiments [24-26] or by isolation of important secondary metabolites that are useful for treating particular types of diseases or disorders [21,27]. In addition, previously published studies can also aid in establishing links between traditional uses and modern scientific knowledge [20,28-31]. The practice of seeking evidence helps in identifying important medicinal plants and may also lead to the development of new or important pharmaceutical drugs [32] with future bioprospecting potential [33,34].

Many ethnopharmacological studies have been conducted in Nepal [15,35-37]; however, many parts of the country and communities remain unexplored. One of the unexplored communities is the Tamang community of the Makawanpur district, which constitutes 47.3% of the total district population [19]. In Nepal, the Tamang community is the fifth largest community, with 5.6% of the total population of the country [19], who mostly live in mountainous or hilly regions [35]. The Makawanpur district is one of the major Tamang dwelling areas in the hilly region of Nepal. Few ethnobotanical studies have been conducted in this district [38-48], although a considerable number of ethnobotanical studies relating to the Tamang community have been conducted in different parts of Nepal [20,22,49-57]. Previous studies in different parts of Nepal have revealed that the Tamang people have a unique culture and a rich traditional knowledge [20,35,55]. However, ethnopharmacological studies specifically targeting the Tamang community of the Makawanpur district are lacking, as are the validation of traditional uses. Therefore, in the present study, we aimed to document indigenous knowledge of the use of medicinal plants in the Tamang community of the Makawanpur district. We hypothesized that the Tamang people in Makawanpur have specialized knowledge of the use of plants, and a wealth of information on a wide range of medicinal plants is expected from the district because the Makawanpur district is located in an area that is rich in biodiversity. We also expected that the Tamang people use different species or known species for different diseases than previously reported because they form part of a distinct ethnic group with a unique identity. Our specific objectives were to address the following questions: (i) Which plant species are used against different diseases by the Tamang people in the Makawanpur district? (ii) What are the modes of preparation and administration of traditional herbal medicines? (iii) Is there any pharmacological or phytochemical evidence for the traditional uses?

## Methods

### Study area

**Figure 1:**
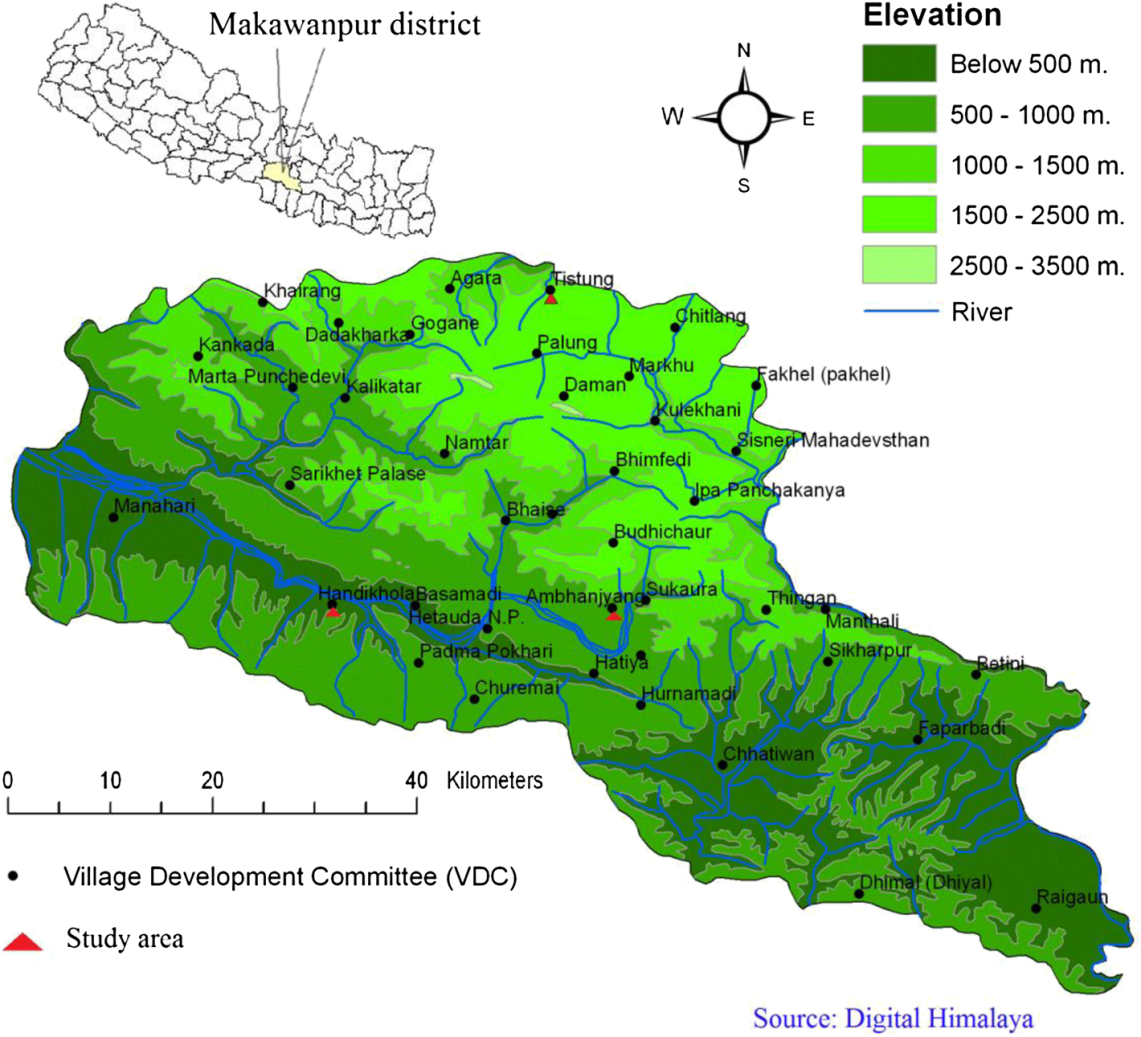
Map Makawanpur district showing study area.

The study areas included the Hadigaun, Aambhanjyang and Tistung village development committees (VDCs) (Figure 1). Study areas were selected based on a large Tamang population residing in the area compared with the population of other communities. Field visits were conducted in March 2011 and May 2011 because it is the peak time for plant growth. The duration of one field visit at a site ranged from 4 to 10 days.

The total number of households in Hadigaun was 3155, with 8900 males and 8970 females. The total number of households in Aambhanjyang was 1519, with 4251 males and 4279 females, and the total number of households in Tistung was 1190, with 3293 males and 3293 females [19]. The types of vegetation differed between sites. In Hadikhola (200–1000 m asl), tropical forest is present, with *Shorea robusta* as the dominant species and common associates of *Terminalia alata, Adina cordifolia, Anogeisus latifolia, Lagerstroemia parviflora, Dillenia pentagyna, Syzygium cumini*, and *Semicarpus anacardium*. There was an *Acacia catechu-Dalbergia sissoo* community along streams and rivers and a tropical deciduous riverine forest with *Bombax ceiba, Holoptelea integrifolia*, and *Trewia nudiflora* together with other species in the *Shorea robusta* forest. In Aambhanjyang (550–1680 m asl), there was subtropical forest with the remains of a *Shorea robusta* forest at lower elevations, and a *Schima-Castanopsis* forest at higher elevations. *Schima wallichii* occurs throughout, with *Castanopsis indica* more common below 1200 m, and *Castanopsis tribuloides* above this elevation. At higher elevations, there was riverine forest with *Toona*, and *Albizzia* species. This forest occurs in narrow strips along streams and is mixed in composition, with *Toona ciliata, Albizzia mollis*, and *Pandanus nepalensis*. In Tistung (1500–2500 m asl), there were pure stands of *Pinus roxburgii* characterized as lower temperate forest type.

### Data collection

Prior to data collection, the objectives of the study were discussed by organizing a group meeting with local people, teachers, elderly men and women from each VDC. Verbal consent was obtained from the participants, and data were collected in a manner similar to other studies in Nepal [58,59].

The participants were chosen to represent both genders and all age groups. We used semi-structured questionnaires in groups in each study area, and questions aimed to collect information on the types of diseases and presence of plant species and their uses. Thirty people in Hadigaun, 17 in Aambhanjyang and 10 in Tistung area participated in the study, of which 22 were women, primarily housewives aged 25–60 years, and 35 were men 40–79 years old (Additional file 1: Table S1). We also conducted separate in-depth interviews with four local healers using structured questionnaires targeting the specific uses of each plant species. The local healers who participated in in-depth interviews included Saila Syantang (70 years old) in Tistung, Lal Bahadhur Thing (68 years old) and Bir Bahadhur Parja (70 years old) in Hadikhola, and Chandra Bahadhur Syantang (50 years old) in the Ambhanjyang VDCs. In addition, we also collected information on veterinary use. The information collected through non-structured questionnaires and structured questionnaires was summarized into a single table providing all plant names, and their uses. The final table included information on vernacular names, life forms, growth, local status, growth ranges, sources, parts used, preparation, administration, and uses. We also included information regarding published studies that cite uses similar to those reported by the Tamang people from the Makawanpur district.

Plant collection and exhibition of plant species were performed during the group or individual interviews. We also asked local people to show plant species obtained outside the Makawanpur district. Most of the collected plants were identified in the field using flora books [60-62]. The identified species were photographed for further references, and unidentified species were preserved as herbarium specimens. The unidentified plant specimens were stored between paper sheets in an herbarium press, and tightened to prevent distortion of the plant. The paper sheets were changed every day for at least three days. The tightly packed herbarium press was kept in sunlight during the daytime to allow evaporation of the moisture to dry the plant specimen, and to prevent infection with fungi using this natural drying technique [63]. The plants were later identified using the same flora books described above and also by comparison with herbarium specimens deposited at the National Herbarium and Plant Laboratories (KATH), Godawari, Lalitpur, Nepal. All specimens collected were deposited at KATH. We followed the nomenclature of Press et al. (2000) [64].

### Comparing traditional knowledge

The uses of plant species described by the Tamang people in the Makawanpur district were compared with several studies related to the Makawanpur district [38-43,45-48] and Tamang communities from different parts of Nepal [20,22,49-57,65]. We also consulted several books that described medicinal plants throughout Nepal. These books were the outcome of studies conducted in different parts of Nepal [35,66] or specific regions [67] or review books on medicinal plants [15,36,68]. We also consulted recent studies that were not included in the books described above [7,17,20,29,30,47,59,69-72]. The pharmacological studies included tests of crude or purified plant extracts against a particular type of microbe or disease. Phytochemical studies included isolation of compound(s), and such compounds were sometimes tested against microbes or diseases. To identify the studies, we searched for relevant literature on plant species in different electronic databases (ISI Web of Science, MEDLINE, Science Direct, Scopus, and Google Scholar) and by searching masters and Ph. D. dissertations at Tribhuvan University Central Library, Kirtipur, Nepal.

## Results

### Medicinal plant diversity, and uses

The present survey found 161 plant species belonging to 86 families and 144 genera that are used to cure 89 human ailments by the Tamang people in the Makawanpur district (Additional file 2: Table S2). Angiosperms constituted the highest number of plants species (132 dicotyledones, and 18 monocotyledones) followed by pteridophytes (6), gymnosperms (3), and mushrooms (1). Herbs were the major sources of medicine (45%), followed by trees (33%), and shrubs (23%).

**Figure 2:**
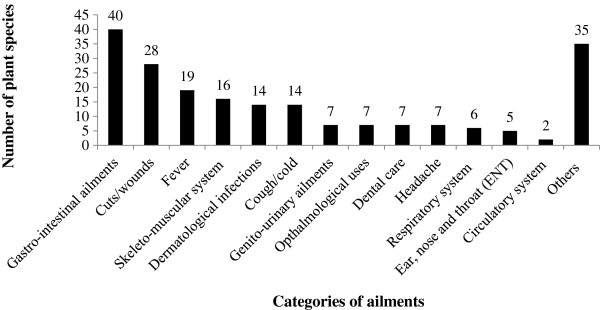
**Use frequency of plant species in different disease categories by Tamang community in Makawanpur district.** The plant species might have repeated for each disease category.

The highest number of plant species was used for gastrointestinal-related diseases followed by cuts and wounds, and fever. Thirty-five plant species were used for other categories (antidotes, improved lactation, cooling agents, tonics, and for religious purposes) (Figure 2).

Some of the plants were edible and were also used as food (*Dryoathyrium boryanum*, *Musa paradisiaca*, *Myrica esculenta*, *Psidium guajava*, and *Rubus ellipticus*). Wide varieties of trees or shrubs were major sources of fuel or sometimes timber for daily uses (e.g., construction, and making wooden tools such as ploughs). Other species were used for religious purposes (e.g., *Shorea robusta*, *Pinus roxburghii*, and *Pinus wallichiana*).

### Parts, preparation, modes of use, and harvesting

**Figure 3:**
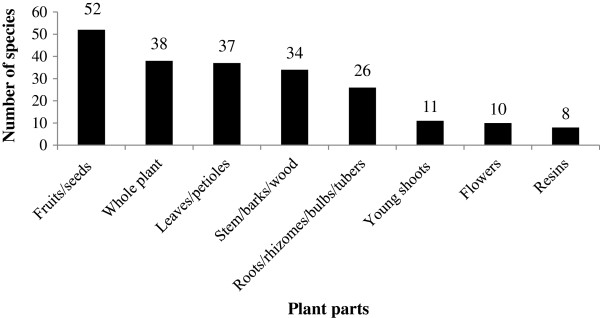
Use frequency of different plant parts by Tamang community in Makawanpur district.

The plant parts used for various types of ailments included the underground parts (roots, rhizomes, bulbs, and tubers), young shoots, stems, bark and wood, leaves and petioles, flowers, fruits and seeds, resins, and the whole plant. The most frequently used plant parts were fruits and seeds (for 51 different plant species), followed by leaves and petioles (37 plant species). The whole plant was also frequently used (38 plant species) (Figure 3).

**Figure 4:**
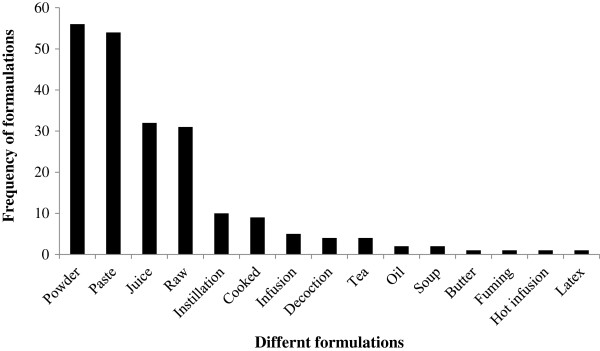
Use frequency of different formulations used adopted by Tamang community in Makawanpur district.

The plants were used in various forms for different ailments. The most frequently used form was powder, followed by paste, and juice. Many species were also used as medicine in a raw form (Figure 4). The primary mode of administration of medicine to cure different ailments was oral (56.47%), followed by external or topical application (40.59%). Three species were used as a toothbrush (*Hedera nepalensis*, *Prunus cornuta*, and *Smilax zeylanica*). *Vitex negundo* was used by fuming and administered through the nasal passage. *Drymaria diandra* was mixed with water for steam therapy.

Plant species were primarily harvested from the wild (147 plant species), and only a few species were cultivated (14 plant species) (Additional file 2: Table S2). Plants were generally collected as per local needs without any rules, and regulations. Four species (*Malus sylvestris*, *Nardostachys grandiflora*, *Pycnoporus cinnabarinus*, and *Valeriana jatamansii*) were obtained from the Rasuwa district, and one species from its only habitat in western Nepal (*Pterocarpus marsupium*) (Additional file 2: Table S2).

### Differences between respondents

During the interviews, we found that elderly people were most familiar with the various types of medicinal plant species. The majority of the participants knew plants used for common ailments such as stomach problems, cough, and cold. When comparing males and females, females were better at identifying plant species than males because they regularly visit forests for firewood or fodder collection. The majority of young people were not familiar with plant species, and their uses. The local healers participated and described the uses of the plants but were reluctant to share their full indigenous knowledge on the uses of plants, and did not reveal the detailed doses of administration. Most of the people reported that traditional knowledge was passed through verbal communication, and repeated practice. The healers reported that knowledge of the use of plants was passed to one of their family members without revealing this knowledge to the others.

### Veterinary medicines

There were 14 plant species belonging to 14 families used for veterinary diseases. Most of these plants were given as the whole plant by oral administration, and a few were used topically. The plants were used for a wide variety of diseases, such as diarrhea, wounds, infections, and foot-and-mouth disease (Table 1).

**Table 1:** Veterinary uses of medicinal plants in Makawanpur area

**Scientific Names**	**Administration**	**Uses**
*Asparagus racemosus* Willd.	Oral	Better milk production
*Auricularia polytricha* (Mont.) Sacc.	Oral	Dyspepsia
*Bauhinia malabarica* Roxb.	Topical	Wounds in neck
*Cannabis sativa* L.	Oral	Diarrhea
*Cissampelos pareira* L.	Oral	To kill tapeworm
*Coriandrum sativum* L.	Oral	Better milk production
*Didymocarpus albicalyx* C. B. Clarke	Oral	Energies the weak animals
*Elephantopus scaber* L.	Oral/Topical	Sex stimulants in female animals
*Lindera neesiana* (Wall. ex Nees) Kurz	Oral	Diarrhea
*Mangifera indica* L.	Topical	Eye problems
*Persicaria chinensis* (L.) H. Gross	Oral	Diuretic
*Viscum album* L.	Oral/Topical	Wide ranges of diseases especially for buffalos
*Zanthoxylum oxyphyllum* Edgew.	Topical	To kill lice and teaks
*Zingiber officinale* Rosc.	Oral/Topical	Foot mouth diseases and other communicable diseases

### Comparison of reported uses

From a literature comparison, we found that there were similar use reports for 93 plant species used against 64 different ailments in various studies. Although 68 plant species were described as medicinal in various studies, 55 different uses by the Tamang people were not found in any of the literature compared. Moreover, five plant species (*Gaultheria hookeri*, *Malvaviscus arboreus*, *Osmanthus suavis*, *Sambucus hookeri*, and *Schoenoplectus juncoides*) were not found as medicinal plant species in any of the studies we considered for comparison (Additional file 2: Table S2).

Pharmacological and phytochemical studies were found for 60 plant species (Additional file 3: Table S3). A comparison of Tamang use with pharmacological and phytochemical studies showed complete or partial consistency for 52 of 60 plant species. The studies for eight plant species (*Astilbe rivularis*, *Citrus aurantifolia*, *Cucumis sativus*, *Eupatorium adenophorum*, *Pinus wallichiana*, *Rhododendron arboreum*, *Scutellaria repens* and *Valeriana jatamansii*) were related to the isolation of different compounds, but the compounds had not been pharmacologically tested against the disease mentioned by Tamangs of Makawanpur (Additional file 3: Table S3).

## Discussion

### Traditional uses of medicinal plants by the Tamang people in Makawanpur

In the present study, we reported 161 plant species that are used by the Tamang people in the Makawanpur district as medicine. The number of reported medicinal plant species is higher than in studies carried out in the Chepang community [45], in various communities [40], in the Daman area in the Makawanpur district [46], and in the Tamang community in the Rasuwa district [20] in Nepal. This observation shows that the Tamang people in the Makawanpur district have extensive knowledge of how to use plants as medicine against different diseases. The dominance of herbs followed by trees, and shrubs is consistent with the different studies from Nepal [7,17,18,20]. The preference for herbs over other forms may be because herbs are more abundant [7], and more easily collected and transported [20].

The most frequently cured disease category was gastrointestinal diseases. Similar to other rural communities, the prevalence of gastrointestinal diseases in the Tamang community is due to poor sanitation, and contaminated drinking water [73]. The plants are used as food, timber and fuel, and also as veterinary medicine in the Makawanpur district, demonstrating that the residents fulfill different requirements from plants as reported in various previous studies [15,35,36].

Fruits and seeds, and leaves and petioles are most frequently used because they are easily available. The preference for fruits and seeds or leaves and petioles for primary health care shows that indigenous knowledge is quite specialized because these parts contain high concentrations of bioactive compounds [74], comparable to underground plant parts [75,76].

### Comparison of reported uses

Although 93 plant species showed similar uses with other studies from different parts of Nepal (Additional file 2: Table S2), we found pharmacological or phytochemical studies for only 60 of these plant species (Additional file 3: Table S3). These 52 medicinal plant species have good bioprospecting potential because they are scientifically proven to be important in cures for different diseases, which demonstrates that the Tamang people in the Makawanpur area have reliable knowledge on the use of plants for their primary health care.

When looking across the different studies, we found that uses for five different plant species were not exactly the same as previously reported, but the previously reported uses were similar. For example, we reported that *Aconitum ferox* was used for toothache, and was described as an analgesic in other studies [15,68]. *Amaranthus spinosus,* which is used against skin diseases in Makawanpur, is reported as being used for wounds [38], to remove pus in boils and for various skin problems such as boils, burns, pimples, and eczema [35]. Likewise, *Artemisia indica,* which is reported to be used for scabies, was previously reported as being used for bathing children [47], lacerations [20] or against wounds or ringworm [35]. *Cassia fistula,* used for digestion problems in the studied area, was previously reported as a laxative or appetite stimulant in Nepal [35,43]. *Cinnamomum tamala,* reported to be beneficial for digestion, was previously described as a carminative [15] or used for diarrhea [35].

Given that we have revealed previously unreported uses for 68 of 161 species, it is important to have explicit documentation of the use of plants in different parts of Nepal so that the valuable but disappearing traditional knowledge will be preserved. Further investigation of plant species related to pharmacological and phytochemical studies may lead to the discovery of new bioactive compounds for treating life-threatening illnesses [77,78].

### Harvesting and sustainable management of medicinal plants

Harvesting of plant species from the wild is a common trend worldwide [79-81]. Seven of the species used by the Tamang people in Makawanpur are protected [82], including *Acacia catechu*, *Bombax ceiba*, *Juglans regia*, *Shorea robusta*, and *Taxus wallichiana*. None of these plant species was traded outside the study area. However, many of these plant species (e.g., *Acacia catechu*, *Acorus calamus*, *Asparagus racemosus* and *Paris polyphylla*) possess the potential to boost the economy in the future [83]. The use of plants from the wild in these regions demonstrates that medicinal plants face the long-term danger of depletion, and therefore, their cultivation should be initiated to save this component of biodiversity, and maintain the existing ecosystems. In addition, populations of many medicinal plant species are often reduced by deforestation, habitat encroachment, shifting cultivation, forest fires, grazing, and other anthropogenic activities. Therefore, cultivation techniques for several medicinal plant species are currently being tested at Daman Botanical Garden and Tistung Botanical Garden (e.g., *Acorus calamus*, *Amomum subulatum, Asparagus racemosus*, *Astible rivularis*, *Berginia ciliata*, *Lobelia pyrimidalis*, and *Mentha piperata*) by the Department of Plant Resources under the Ministry of Forest and Soil Conservation, Nepal. Knowledge of the cultivation techniques required should be transferred to the local farmers in the study area.

## Conclusions

The Tamang community in the Makawanpur district has rich indigenous knowledge of the use of medicinal plants to maintain their primary health. Traditional herbal remedies are important and effective in the Tamang community because many traditional uses are scientifically proven through phytochemical and pharmacological studies. However, a large number of plant species remain untested for bio-efficacy and toxicity. Such tests may reveal novel remedies that have bioprospecting potential. Most of the plant species are harvested in the wild; the practice of cultivation and domestication of at least the most rare and most highly used plant species is needed for sustainability.

## Competing interests

The authors declare that they have no competing interests.

## Authors’ contributions

DL carried out field research. MR supervised the work. DL, MR, BT and ZM analysed the data and wrote the manuscript. All authors approved the final version of the manuscript.

## Supplementary Material

Additional file 1: Table S1 Details of informants interviewed in Hadigaun, Aambhanjyang and Tistung village development committees of Makawanpur district. Click here for file

Additional file 2: Table S2 Medicinal plants used by Tamang community in Makawanpur district, central Nepal [38,84-93]. Click here for file

Additional file 3: Table S3 Comparison of local use and phytochemical and pharmacological studies of medicinal plants [94-186]. Click here for file
