# Supplementary material for: Medicinal plants used by the Tamang community in the Makawanpur district of central Nepal
Source: J Ethnobiol Ethnomed. 2014 Jan 10;10:5. doi: 10.1186/1746-4269-10-5 (PMC3904474; doi:10.1186/1746-4269-10-5)
Supplement: Additional file 1: Table S1 — Details of informants interviewed in Hadigaun, Aambhanjyang and Tistung village development committees of Makawanpur district. [file 1746-4269-10-5-S1.docx]

**Additional file 1: Table S1** Details of informants interviewed in Hadigaun, Aambhanjyang and Tistung village development committees of Makawanpur district.

| SN | Name | Age | Sex | Address | Occupation |
| --- | --- | --- | --- | --- | --- |
|  | Hadikhola – 7 , Chuwarpakha |  |  |  |  |
| 1 | Lal Bahadur Things | 68 | M | Hadikhola-7 | Local healer, Farmer |
| 2 | Bir Bahadur Parja | 70 | M | Hadikhola-7 | Local healer, Farmer |
| 3 | Mangal Bahadur Moktan | 41 | M | Hadikhola- 7 | Plants collector, Farmer |
| 4 | Kumari Waiba | 35 | F | Hadikhola- 7 | Fodder collector, Farmer |
| 5 | Ful Maya Gole | 26 | F | Hadikhola- 7 | Fodder collector, Farmer |
| 6 | Kumari Bk | 54 | F | Hadikhola- 7 | Fodder collector, Farmer |
| 7 | Som Bahadhur Moktan | 52 | M | Hadikhola- 7 | Fodder collector, Farmer |
| 8 | Ram Bahadhur Moktan | 53 | F | Hadikhola- 7 | Plant collector, Social worker, Farmer |
| 9 | Hari Krishan Gole | 42 | M | Hadikhola- 7 | Social worker, Farmer |
| 10 | Buddhi Bahadhur Titung | 60 | M | Hadikhola- 7 | Knowledgeable person |
| 11 | Maina Bk | 34 | F | Hadikhola- 7 | Fodder collector, Farmer |
| 12 | Srijana Bk | 28 | F | Hadikhola- 7 | Fodder collector, Farmer |
| 13 | Parbat Bahadhur Bk | 45 | M | Hadikhola- 7 | Social worker |
| 14 | Fulman Waiba | 45 | F | Hadikhola- 7 | Social worker ,Farmer |
| 15 | Lal maya Waiba | 40 | F | Hadikhola- 7 | Fodder collector, Farmer |
| 16 | Thulo kancha Moktan | 52 | M | Hadikhola- 7 | Fodder collector, Farmer |
| 17 | Putali Maya Moktan | 60 | F | Hadikhola- 7 | Farmer |
| 18 | Dolma Waiba | 79 | M | Hadikhola- 7 | Farmer |
| 19 | Binda Ghalan | 48 | M | Hadikhola- 7 | Farmer |
| 20 | Sanchuri Chepang | 60 | F | Hadikhola- 7 | Farmer |
| 21 | Mohan parja | 40 | M | Hadikhola- 7 | Farmer |
| 22 | Maili maya Thing | 26 | F | Hadikhola- 7 | Farmer |
| 23 | Kanchi Maya Bal | 25 | F | Hadikhola- 7 | Farmer |
| 24 | Bishnu Thing | 46 | M | Hadikhola- 7 | Farmer |
| 25 | Kanchi Maya Chepang | 45 | F | Hadikhola- 7 | Farmer |
| 26 | Hari Titung | 60 | M | Hadikhola- 7 | Farmer |
| 27 | Jit Bahadhur Thing | 40 | F | Hadikhola- 7 | Farmer |
| 28 | Jiban Waiba | 50 | M | Hadikhola- 7 | Farmer |
| 29 | Somaraj Thing | 49 | M | Hadikhola- 7 | Farmer |
| 30 | Ramsing Waiba | 56 | M | Hadikhola- 7 | Farmer |
|  | Aambhanjyang – 5, Makawanpur | |  |  |  |
| 1 | Chandra Bahadhur Syantang | 50 | M | Aambhanjyang- 5 | Local Healer, Farmer |
| 2 | Laxman Bajgai | 45 | M | Aambhanjyang- 5 | Social worker, Farmer |
| 3 | Isan Bajagai | 55 | M | Aambhanjyang- 5 | Social worker, Farmer |
| 4 | Kumari Syantang | 60 | F | Aambhanjyang- 5 | Knowledgeable person, Farmer |
| 5 | Sanu Maya Thing | 56 | F | Aambhanjyang- 5 | Fodder collector, Farmer |
| 6 | Bikram Bal | 51 | M | Aambhanjyang- 5 | Gardener,Farmer |
| 7 | Bijay Titung | 49 | M | Aambhanjyang- 5 | Local leader, |
| 8 | Purna Bahadhur Thing | 67 | M | Aambhanjyang- 5 | Helper in Garden |
| 9 | Santoshi Syangtang | 38 | F | Aambhanjyang- 5 | Fodder collector,Farmer |
| 10 | Harenanda Bhandari | 63 | M | Aambhanjyang- 5 | Farmer |
| 11 | Thulo kancha Bal | 66 | M | Aambhanjyang- 5 | Farmer |
| 12 | Pabitra Syangtang | 35 | F | Aambhanjyang- 5 | Farmer |
| 13 | Purna Maya Thing | 40 | F | Aambhanjyang- 5 | Farmer |
| 14 | Jit Bahadhur Gole | 49 | M | Aambhanjyang- 5 | Social worker, Farmer |
| 15 | Maili Maya Syangtang | 55 | F | Aambhanjyang- 5 | Fodder collector, Farmer |
| 16 | Saili Gole | 54 | F | Aambhanjyang- 5 | Fodder collector, Farmer |
| 17 | Hasta Bahadhur Thing | 50 | M | Aambhanjyang- 5 | Knowledgeable person, Farmer |
|  | Tistung- 1, Makawanpur |  |  |  |  |
| 1 | Saila Syantang | 70 | M | Tistung- 1 | Local healer |
| 2 | Gopal Karki | 41 | M | Bajrabarahi- 6 | Gardener |
| 3 | Buddi Bahadhur Syantang | 50 | M | Tistung- 1 | Helper in Garden |
| 4 | Sunder Pulami | 55 | M | Tistung- 1 | Knowledgeable person, Farmer |
| 5 | Saili Maya Bal | 56 | F | Bajrabarahi-6 | Fodder collector |
| 6 | Laxmi Nanda Titung | 54 | M | Tistung- 1 | Farmer |
| 7 | Mohan Syantang | 48 | M | Tistung- 1 | Farmer |
| 8 | Karna Bahadur Titung | 48 | M | Tistung- 1 | Social worker |
| 9 | Hemraj Gole | 44 | M | Tistung- 1 | Fodder collector, Farmer |
| 10 | Tapindra Syangtang | 57 | M | Tistung- 1 | Farmer |

SN, serial number; Y,years; M, male; F, female.
