# Supplementary material for: Medicinal plants used by the Tamang community in the Makawanpur district of central Nepal
Source: J Ethnobiol Ethnomed. 2014 Jan 10;10:5. doi: 10.1186/1746-4269-10-5 (PMC3904474; doi:10.1186/1746-4269-10-5)
Supplement: Additional file 2: Table S2 — Medicinal plants used by Tamang community in Makawanpur district, central Nepal [38,84-93]. [file 1746-4269-10-5-S2.docx]

**Additional file 2: Table S2** Medicinal plants used by Tamang community in Makawanpur district, central Nepal [84-94].

| **Scientific name (Family) voucher number** | **^A^Vernacular Names** | **^B^Life forms: Origin** | **Minimum-Maximum altitudinal ranges (meters)** | **Status; Source-Localities** | **^C^Parts used** | **Preparation** | **Application** | **^D^Reported uses** | **Similar uses** |
| --- | --- | --- | --- | --- | --- | --- | --- | --- | --- |
| *Acacia catechu* (L. f.) Willd. (Fabaceae) DLH 151 | Khair (Np), Khair (Tam), Catechu tree (Eng) | Tr: Wil | 200-1400 | Common; Within district - Hadikhola | St/Br/Wd | Paste/Decoction | Topical/Oral | Joints aches, Fever | [35] |
| *Acacia pennata* (L.) Willd. (Fabaceae) DLH 154 | Arerikanda (Np), Kekru (Tam), Wattle plants (Eng) | Tr: Wil | 200-1100 | Common; Within district - Hadikhola | Res | Paste | Oral | Wounds | [35] |
| *Achyranthes bidentata* Blume (Amaranthaceae) DLP 21 | Dattiwan (Np), Phrekhrek (Tam) , Hill chaff flower (Eng) | Hb: Wil | 200-2100 | Common; Within district - Hadikhola,Aambhanjyang, Palung | Ysh | Decoction | Oral | Stomatitis, Common cold ^NU^ |  |
| *Aconitum ferox* Wall. ex Ser. (Ranunculaceae) DLP25 | Bisma (Np), Bingkha (Tam), Acotine (Eng) | Hb: Wil | 2100-3800 | Rare; Inside/Outside district - Simbhanjyang (Makawanpur), Rasuwa | Lvs/pt | Raw | Oral | Toothaches | [15, 68] |
| *Acorus calamus* L. (Araceae) DLP 57 | Bhojo (NP), Seda (Tam), Sweet flag (Eng) | Hb: Cul | 100-2300 | Common; Within district - Hadikhola | Rt | Raw | Oral | Cough, Fever | [15, 20, 47, 68, 84] |
| *Aegle marmelos* (L.) Correa (Rutaceae) DLH 107 | Bel (Np), Bel (Tam), Bail fruit tree (Eng) | Tr: Wil | 600-1100 | Common; Within district - Hadikhola, Aambhanjyang | Fr | Juice/Raw | Oral | Dysentery, Diarrhoea | [15, 35, 36, 45, 47, 68, 84] |
| *Aesandra butyracea* (Roxb.) Baehni (Sapotaceae) DLP58 | Chiuri (Np), Chiuri (Tam), Nepal butter fruit (Eng) | Tr: Wil | 200-1500 | Common; Within district - Hadikhola, Aambhanjyang | Fl | Oil/Butter | Topical | Wounds, Cracked feets ^NU^ |  |
| *Allium wallichii* Kunth (Amaryllidaceae) DLP 92 | Banlasun (Np), Dundudhap (Tam), Wild garlic (Eng) | Hb: Wil | 2400-4650 | Rare; within/outside district - Tistung, Palung (Makawanpur), Kafre (Sindhupalchok), Rasuwa | Bul | Paste | Topical | Wounds, Mumps ^NU^ |  |
| *Aloe vera* (L.) Burm. f. (Liliaceae) DLP 37 | Ghiukumari (Np), Ghyukumari (Tam), Aloe (Eng) | Hb: Cul | 1200-1400 | Common; Within district - Hetauda area | Lvs/pt | Juice | Topical | Fire burns | [35] |
| *Alstonia scholaris* (L.) R. Br. (Apocynaceae) DLP40 | Chhatiwan (Np), Chhatiwan (Tam), Devil's tree (Eng) | Tr: Wil | 100-300 | Common; Within district - Chatiwan, Hadikhola | St/Br/Wd | Powder | Oral | Abortions ^NU^ |  |
| *Amaranthus spinosus* L. (Amaranthaceae) DLP 45 | Ludekanda (Np), Bhagani dhap (Tam), Prickly amaranth (Eng) | Hb: Wil | 150-1200 | Common; Within district - Hadikhola, Aambhanjyang | Rt, Wh pl | Paste | Topical | Skin diseases | [15, 36, 68, 84] |
| *Ammannia auriculata* Willd. (Lythraceae) DLP205 | Jaraeu (Np), Jaraeu (Tam) | Sh: Wil | 700-1400 | Common; Within district - Bajrabarahi, Palung | Rt | Powder | Oral | Malarial fever | [45] |
| *Ananas comosus* (L.) Merr. (Bromeliaceae) DLH 95 | Bhui katahar (Np), Sa katahar ( Tam), Pineapple (Eng) | Hb: Wil | 100-1200 | Common; Within district - Aambhanjyang | Fl | Raw/Juice | Oral | Fever |  |
| *Artemisia indica* Willd. (Asteraceae) DLP 67 | Tite pati (Np), Chandre (Tam), Mugwort (Eng) | Hb: Wil | 300-2400 | Common; Within district - everywhere | Wh pl | Paste/Juice | Topical | Scabies | [15, 35, 68] |
| *Artocarpus heterophyllus* Lam. (Moraceae) DLP 98 | Katahar(Np), Sing katahar(Tam), Jack Fruit (Eng) | Tr: Cul | 800 | Common; Within district - everywhere | Fr/Sd | Paste/Juice | Topical | Boils, Bruises | [15, 85, 86] |
| *Artocarpus lakoocha* Wall. ex Roxb. (Moraceae) DLP105 | Badahar (Np), Sing barahar (Tam), Monkey jack (Eng) | Tr: Wil | 100-1300(-1800) | Common; Within district - everywhere | Fr/Sd | Paste/Juice | Topical | Boils, Bruises | [35] |
| *Asparagus racemosus* Willd. (Asparagaceae ) DLH111 | Kurilo (NP), Komo (Tam), Wild asparagus (Eng) | Sh: Cul | 300-2200 | Common; Within district - Tistung, Palug, Hadikhola | Rt | Cooked | Oral | Better lactation | [35, 36, 68] |
| *Astilbe rivularis* Buch.-Ham. ex D. Don (Saxifragaceae) DLP35 | Thulo okhati (Np), Ganchhyug mran (Tam) | Hb: Wil | 2000-3600 | Rare; Within district - Daman | St/Br, Lvs | Powder/Juice | Oral | Energizer for delivered women | [40, 46] |
| *Azadirachta indica* A. Juss. (Meliaceae) DLP 65 | Neem (Np), Nim (Tam), Margosa tree (Eng) | Tr: Wil | 900 | Common; Within district - Hetauda area | Br/st, Lvs | Paste/Juice | Topical | Scabies, Skin diseases | [35] |
| *Bauhinia vahlii* Wight & Arn. (Fabaceae) DLH 69 | Vorla (Np), Bhorla (Tam), Camel’s foot climber (Eng) | Tr: Wil | 200-1300 | Common; Within district - Hadikhola, Aambhanjyang | Rt, Fl | Paste/Powder | Topical/Oral | Paralysis, Diarrhoea | [35] |
| *Bauhinia variegata* L. (Fabaceae) DLH66 | Koiralo (Np), Aanbu (Tam), Orchid tree (Eng) | Tr: Wil | 150-1900 | Common; Within district - Hadikhola, Aambhanjyang | Fr | Powder/Cooked | Oral | Dysentery | [15, 68, 84, 85] |
| *Benincasa hispida* (Thunb.) Cogn. (Menorrhagea) DLP 87 | Kuvindo (Np), Kuvindo (Tam), White guard (Eng) | Hb: Cul | 1200-1400 | Common; Within district - Hadikhola, Aambhanjyang | Fr | Powder/Cooked | Oral | Abortions ^NU^ |  |
| *Berberis aristata* DC. (Berberidaceae) DLP85 | Chutro (Np), Yamjuki jungba (Tam), Barberry (Eng) | Sh: Wil | 1600-3500 | Common; Within district - Aambhanjyang, Palung, Hatiya | Br/St/Wd | Instillation | Topical | Eye infection | [15, 35, 46, 68, 84, 85] |
| *Bergenia ciliata* (Haw.) Sternb. (Saxifragaceae) DLP60 | Pakhan Ved (Np), Brasen (Tam), Rock foil (Eng) | Hb: Wil | 1300-3200 | Common; Within district - Aghor, Simbhanjyang,Bajrabarahi, Tistung | St/Pt | Juice/Powder | Oral | Energizer for delivered women | [40] |
| *Blumea balsamifera* (L.) DC. (Asteraceae) DLP69 | Gaitihare (Np), Gaitihare (Tam) | Sh: Wil | 500 | Common; Within district - Tistung, Sarikhet, | Lvs/Fr | Powder | Oral | To relieve hotness | [15, 85, 87] |
| *Bombax ceiba* L. (Bombacaceae) DLP164 | Simal (Np), Kagdhong (Tam), Silk cotton tree (Eng) | Tr: Wil | 200-1200 | Common; Within district - Hadikhola | St/Lvs/Fl/Res | Paste | Topical | Fire burns ^NU^ |  |
| *Buddleja asiatica* Lour. (Loganiaceae) DLP187 | Bhimsenpati (Np), Bhisa (Tam), Butterfly bush (Eng) | Tr: Wil | 350-2000 | Common; Within district - Bajrabarahi, Palung | St/Lvs | Juice | Topical | Wounds, For worshipping | [15, 35, 84] |
| *Butea minor* Buch.-Ham. ex Baker (Fabaceae) DLP169 | Bhuletro (Np) | Sh: Wil | 300-2000 | Common; Within district - Hadikhola | Fr/Sd | Juice/Powder | Oral | To expel round worms | [35, 38, 45] |
| *Callicarpa arborea* Roxb. (Verbinaceae) DLP132 | Guyalo (Np), Goldar (Tam) | Tr: Wil | 250-2000 | Common; Within district - everywhere | Br/Lvs | Juice/Powder | Oral | Stimulants | [35] |
| *Cannabis sativa* L. (Cannabaceae) DLP131 | Ganja (Np), Wang/Sima (Tam), Hemp (Eng) | Hb: Wil | 200-2700 | Common; Within district - everywhere | Fr/Sd | Powder/Latex | Oral | Diarrhea | [15, 20, 35, 38, 45, 68, 85] |
| *Cassia fistula* L. (Fabaceae) DLP119 | Rajbrikcha (Np), Gle mhendo (Tam), Drum stick (Eng) | Tr: Wil | 150-1400 | Common; Within district - Hadikhola | Rt/Fr/Sd | Powder | Oral | Digestion , Improve urinary flow | [15, 35, 36, 68, 84, 85] |
| *Cautleya spicata* (Sm.) Baker (Zingiberaceae) DLH106 | Panisarro (Np), Panisaro ( Tam) | Hb: Wil | 1800-2800 | Rare; Within district - Tistung, Palung | Lvs | Instillation | Topical | Eye troubles, Conjuctivitis ^NU^ |  |
| *Centella asiatica* (L.) Urb. (Apiaceae) DLH 109 | Ghodtapre(Np), Ghortapre (Tam), Indian pannywort (Eng) | Hb: Wil | 500-2100 | Common; Within district - everywhere | Res | Juice/Paste | Oral/Topical | Purification and retention of urine, Headache | [15, 35, 36, 38, 40, 68, 84] |
| *Cheilanthes bicolor* (Forssk.) Kaulf. (Pteridaceae) DLP152 | Kalisinki(Np), Damkans (Tam) | Hb: Wil | 500-1500 | Common; Within district - everywhere | Lvs/Pt/Wl Pl | Powder | Oral/Topical | Sinusitis, Fever, Cuts ^NU^ |  |
| *Chesneya nubigena* (D. Don) Ali (Fabaceae) DLH161 | Chyali (Np), Chyauli (Tam) | Sh: Wil | 3600-5200 | Common; Within district - simbhanjyang | Lvs/Pt/Wl Pl | Powder | Oral | To regulate menusration cycle ^NU^ |  |
| *Cinnamomum camphora* (L.) J. Presl (Lauraceae) DLP149 | Kapur (Np), Kapur (Tam), Camphor tree (Eng) | Tr: Wil | 1300-1500 | Rare; Within district - Hadikhola | Lvs/Fr/Sd | Raw | Oral | Dental diseases ^NU^ |  |
| *Cinnamomum tamala* (Buch.-Ham.) Nees & Eberm. (Lauraceae) DLP12 | Sinkauli (Np), Dalchini/ Lepte (Tam), Nepalese cinnamon (Eng) | Tr: Wil | 450-2000 | Rare; Within district - Bajrabarahi, Palung | Br/Lvs | Cooked | Oral | Spices benifical for digestion | [15, 68, 88] |
| *Citrus aurantifolia* (Christ.) Swingle (Rutaceae) DLP99 | Kagati (Np), Kaagat (Tam), Lime (Eng) | Tr: Cul | 1400 | Common; Within district - Hadikhola, Aambhanjyang | Fr | Instillation | Topical | Wounds in eyes | [47] |
| *Citrus limon* (L.) Burm. f. (Rutaceae) DLP100 | Nibuwa (Np), Nibuwa ( Tam), Lemon (Eng) | Tr: Cul | 1600 | Common; Within district - Tistung, Hadikhola, Aambhanjyang | Fr | Juice | Oral | Cholera ^NU^ |  |
| *Corydalis chaerophylla* DC. (Papavaraceae) DLP155 | Pahele (Np), Pile (Tam) | Hb: Wil | 1500-4500 | Common; Within district - Tistung | Lvs/Fl | Powder | Oral | Tonic for weakness, In worshipping ^NU^ |  |
| *Cucumis sativus* L. (Cucurbitaceae) DLP164 | Kankro (Np), Langai (Tam), Cucumber (Eng) | Hb: Wil | 1600 | Common; Within district - everywhere | Ysh/Fr | Paste | Topical | Snake bite ^NU^ |  |
| *Curcuma angustifolia* Roxb. (Zingiberaceae) DLH16 | Haledo (Np), Haledo (Tam), Termeric (Eng) | Hb: Cul | 1500 | Common; Within district - everywhere | Rh | Powder | Oral | Stomach problems, Indigestions | [15, 36, 68] |
| *Curcuma aromatica* Salisb. (Zingiberaceae) DLH27 | Banhaledo (Np), Haldi (Tam), Turmeric (Eng) | Hb: Wil | 700-1100 | Rare; Within district - Daman | Rh | Powder | Oral | Stomach problems | [15, 85] |
| *Cuscuta reflexa* Roxb. (Convulvulaceae) DLH73 | Akashbeli (Np), Akasbeli (Tam), Dodder (Eng) | Hb: Wil | 500-3000 | Common; Within district - everywhere | Wh pl | Decoction | Oral | Jaundice | [15, 35, 36, 84] |
| *Cynodon dactylon* (L.) Pers. (Poaceae) DLH72 | Sete dubo (Np), Tabanh (Tam), Bahama grass (Eng) | Hb: Wil | 100-3000 | Common; Within district - everywhere | Wh pl | Juice | Topical | Skin wounds | [15, 35, 68, 85] |
| *Cyperus rotundus* L. (Cyperaceae) DLH191 | Mothe (Np), Gla-sgang (Tam), Nut grass (Eng) | Hb: Wil | 300-2400 | Common; Within district - everywhere | Rt | Juice | Oral | Vomiting | [15, 85] |
| *Datura metel* L. (Solanaceae) DLH182 | Kalo Dhaturo (Np), Dhatura bish (Tam), Dawny datura (Eng) | Hb: Wil | 300-1200 | Common; Within district - Hetauda area | Rt, Lvs, Fr | Powder | Oral | Anti-rabies | [15, 36, 68, 84, 85] |
| *Daucus carota* L. (Apiaceae) DLH177 | Jangali gajar (Np), Gajjar (Tam), Carrot (Eng) | Hb: Wil | 500-3000 | Rare; Within district - Bajrabarahi, Palung | Rt | Powder | Oral | Fever ^NU^ |  |
| *Dendrocalamus* *hamiltonii* Nees & Arn. ex Munro (Gramineae) DLH170 | Bans (Np), Tama bans (Tam) | Tr: Wil | 500-2000 | Common; Within district - Hadikhola | St | Powder | Oral | Typhoid ^NU^ |  |
| *Desmodium multiflorum* DC. (Fabaceae) DLP401 | Bakhri Ghans (Np), Saritambo ghugi (Tam) | Sh: Wil | 1800-2600 | Rare; Within district - Tistung | Rt/Lvs/Br | Powder | Oral | Typhoid |  |
| *Dillenia pentagyna* Roxb. (Dilleniaceae) DLP155 | Tatari (Np), Tatari (Tam) | Tr: Wil | 150-1500 | Common; Within district - Hadikhola, Aambhanjyang, Tistung | St/Lvs | Paste | Topical | Scorpion sting | [35, 43] |
| *Dioscorea alata* L. (Dioscoreaceae) DLH192 | Ghar Tarul (Np), Rhideme temme (Tam), Yam (Eng) | Hb: Wil | 600-1200 | Common; Within district - everywhere | Rh | Cooked/Powder | Oral | To neutralize mushroom poisoning ^NU^ |  |
| *Drymaria diandra* Blume (Caryophyllaceae) DLP411 | Avijalo (Np), Lata pate chhe (Tam) | Hb: Wil | 700-2000 | Common; Within district - everywhere | Wh pl | Raw | Steam inhalation | Sinusitis | [15, 35] |
| *Dryoathyrium boryanum* (Willd.) Ching (Woodsiaceae) DLP406 | Kalo neuro (Np), Dunde (Tam) | Hb: Wil | 1400-3000 | Common; Within district - everywhere | Pt | Cooked | Oral | Cooling agent, Vegetables | [35] |
| *Elaeagnus* *infundibularis* Momiy. (Elaegnaceae) DLP429 | Goyalo (Np), Guaenlo (Tam), Bastard oleaster (Eng) | Sh: Wil | 1500-2500 | Common; Within district - everywhere | St/Br/Fr/Sd | Paste | Topical | Boils ^NU^ |  |
| *Elaeocarpus sphaericus* (Gaertn.) K. Schum. (Elaeocarpaceae) DLP451 | Rudrakshaya (Np), Rudrakshaya (Tam), Utrasum bead tree (Eng) | Tr: Wil | 700-1700 | rare; Within district - Aghor, Simbhanjyang,Bajrabarahi | Sd | Paste | Oral | Cough especiall for children, Worshipping | [15, 68] |
| *Eleusine coracana* (L.) Gaertn. (Poaceae) DLP490 | Kodo (Np), Sangna (Tam), Millet (Eng) | Hb: Cul | 1100-2000 | Common; Within district - everywhere | Sd | Paste | Topical | Wounds | [35] |
| *Elsholtzia flava* (Benth.) Benth. (Lamiaceae) DLP263 | Bansilam (Np), Palunget (Tam) | Hb: Wil | 1900-2700 | Rare; Within district - Sarikhet, Raksirang | Sd/Wh pl | Oil/Juice | Topical | Foot wounds, Foot cracks ^NU^ |  |
| *Engelhardia spicata* Lesch. ex Blume (Juglandaceae) DLP203 | Mauwa (Np), Gundu (Tam) | Tr: Wil | 500-2000 | Common; Within district - Tistung | Fl | Raw | Oral | Abdominal pain ^NU^ |  |
| *Entada phaseoloides* (L.) Merr. (Fabaceae) DLP205 | Lekh Pangra (Np), Lekha pangra (Tam), Nicker bean (Eng) | Sh: Wil | 350-1600 | Rare; Within district - Daman, Simbhanjyang | Sd | Powder | Oral | Constipation, Iron deficinecy ^NU^ |  |
| *Entada rheedei* Spreng. (Fabaceae) DLH86 | Pangro (Np), Pangra (Tam) | Sh: Wil | 350-1600 | Rare; Within district - Daman, Aambhanjyang | Sd | Powder | Oral | Iron deficiency, Bone pain | [20] |
| *Eupatorium adenophorum* Spreng. (Asteraceae) DLP93 | Banmara(Np), Kurum (Tam), Crofton weed (Eng) | Hb: Wil | 500-2400 | Common; Within district - everywhere | Lvs | Juice | Topical | To control bleeding during cuts in skin | [20, 35, 40, 43, 46] |
| *Euphorbia hirta* L. (Euporbiaceae) DLP94 | Dhudhe Jhar (Np), Chumen ( Tam), Asthama weed (Eng) | Hb: Wil | 150-1500 | Common; Within district - everywhere | Wh pl | Juice/Paste | Topical | Cuts, Snake bite | [35] |
| *Euphorbia royleana* Boiss. (Euphorbiaceae) DLP95 | Siudi (Np), Desya (Tam), Cattus spurge (Eng) | Hb: Wil | 1100-1200 | Common; Within district - everywhere | Wh pl | Powder | Oral | Constipation | [15, 35, 39, 40, 84, 89–91] |
| *Ficus racemosa* L. (Moraceae) DLP610 | Dumri (Np), Dumri (Tam), Cluster fig (Eng) | Tr: Wil | 300 | Common; Within district - everywhere | St/Br/Fr | Powder | Oral | Diarrhea | [35] |
| *Ficus religiosa* L. (Moraceae) DLP236 | Pipal (Np), Papal (Tam), Peepal tree( Eng) | Tr: Wil | 150-1500 | Common; Within district - everywhere | St/Br/Fr | Instillation | Topical | Ear infection and pain | [40] |
| *Ficus semicordata* Buch.-Ham. ex Sm. (Moraceae) DLP205 | Khaniya (Np), Kosing (Tam) | Tr: Wil | 200-1700 | Common; Within district - everywhere | St/Br/Fr | Powder/Paste | Topical | Wounds ^NU^ |  |
| *Fragaria daltoniana* J. Gay (Rosaceae) DLP227 | Bhuin Kaphal, Bhuin Aiselu (Np) | Hb: Wil | 2000-2800 | Common; Within district - Daman | Wh pl | Juice | Topical | Wounds, Cuts ^NU^ |  |
| *Fragaria nubicola* Lindl. ex Lacaita (Rosaceae) DLP486 | Bhuin Aiselu (Np), Indian strawberry (Eng) | Hb: Wil | 1600-4000 | Rare; Within district - Daman, Simbhanjyang | Fr | Raw | Oral | Throat problems ^NU^ |  |
| *Gaultheria fragrantissima* Wall. (Ericaceae) SLP409 | Dhasingre (Np), Chinso (Tam), White heater (Eng) | Sh: Wil | 1200-2600 | Common; Within district - Tistung, Simbhanjyang | Fr | Paste | Topical | Wounds, Bone Fractures ^NU^ |  |
| *Gaultheria hookeri* C. B. Clarke (Ericaceae) DLP416 | Patpate (Np), Chenjuwa (Tam) | Sh: Wil | 3200-3500 | Rare; Within district - Tistung, Daman | St/Br/Lvs/Pet/Wh pl | Paste | Topical | Wounds ^NU^ |  |
| *Girardinia diversifolia* (Link) Friis (Urticaceae) DLP452 | Allo, Allo sisnu (Np), Pachyar (Tam), Himalayan nettle (Eng) | Hb: Wil | 1700-3000 | Common; Within district - Raksirang, Sarikhet | Rt | Powder | Topical | Carbuncle | [35] |
| *Glycyrrhiza glabra* L. (Fabaceae) DLP36 | Jethimathu (Np), Jethimadhu (Tam) | Hb: Wil | < 1000 | Rare; Within district/Outside - Tistung, Daman | Wh pl | Cooked | Oral | Gastritis |  |
| *Gmelina arborea* Roxb. (Verbinaceae) DLP466 | Khamari (Np), Khamari(Tam) | Tr: Wil | 200-1100 | Common; Within district - manahari | St/Lvs | Raw | Oral | Tonic ^NU^ |  |
| *Guizotia abyssinica* (L. f.) Cass. (Asteraceae) DLP469 | Jhuse til(Np), Chadong / Sa (Tam), Niger seed (Eng) | Hb: Wil | 900-1900 | Rare; Within district - Hadikhola, Hatiya | Fr | Paste/Instillation | Topical | Wounds, Earache | [35] |
| *Hedera nepalensis* K. Koch (Aralianceae) DLP48 | Kathe laharo, Dudelaa (Np) , Tingali (Tam) | Sh: Wil | 2000-3200 | Common; Within district - Simbhanjyang | St/Lvs | Raw | Toothbrush | Stomatitis | [15, 35, 68, 84, 92] |
| *Imperata cylindrica* (L.) P. Beauv. (Gramineae ) DLP63 | Siru (Np), Sirru (Tam), Cogos grass ( Eng) | Hb: Wil | < 2400 | Common; Within district - everywhere | Rt | Paste | Topical | Headache ^NU^ |  |
| *Indigofera pulchella* Roxb. (Fabaceae) DLP59 | Mirmire (Np), Chisro (Tam) | Sh: Wil | 300-1700 | Common; Within district - Aghor, Simbhanjyang, Bajrabarahi | Lvs | Powder | Oral | Malarial fever ^NU^ |  |
| *Jatropha curcas* L. (Euphorbiaceae) DLH107 | Sajuwan (Np), Desya (Tam), Physic nut (Eng) | Sh: Wil | 500-1200 | Common; Within district - Hadikhola, Padampokhari | Ysh | Paste/Juice | Topical | Wounds in the feet during summer due to mud | [15, 35, 85] |
| *Juglans regia* L. (Juglandaceae) DLH111 | Okhar (Np), Kato (Tam), Walnut (Eng) | Tr: Wil | 1200-3000 | Common; Within district - Bajrabarahi, Palung | Rt/Br | Paste, raw/powder | Topical, oral | Wounds, Tooth ache | [15, 40, 68, 84, 85, 93] |
| *Justicia adhatoda* L. (Acanthaceae) DLP447 | Asuro, Vashak(Np), Basak(Tam), Malabar nut (Eng) | Sh: Wil | 500-1600 | Common; Within district - Aghor, Bajrabarahi | Wh pl | Juice | Oral | Cough, Fever | [35, 40, 47] |
| *Lawsonia inermis* L. (Lytharaceae) DLH67 | Meharee(Np), Meharee (Tam) | Sh: Wil | 500 | Rare; Within district - Rasuwa | Fr/Sd | Paste | Topical | To control hairfall and dendruf with small pinpleson head | [15, 85, 86] |
| *Lecanthus peduncularis* (Royle) Wedd. (Urticaceae) DLP53 | Kholejhar (Np), Tilo (Tam) | Hb: Wil | 1200-3200 | Common; Within district - Palung | Wh pl | Powder | Oral | Asthma, Fever | [15, 35] |
| *Lepidium sativum* L. (Brassicaceae) DLH52 | Chamsur (Np), Chamsur dhap (Tam), Garden cress (Eng) | Hb: Cul | 200-3000 | Common; Within district - everywhere | Wh pl | Cooked | Oral | Rheumatic pain ^NU^ |  |
| *Leptodermis lanceolata* Wall. (Rubiaceae) DLP268 | Bhui champa(Np), Rumsing (Tam) | Sh: Wil | 2000-3500 | Common; Within district - Tistung | Wh pl | Paste | Topical | Fractures bones ^NU^ |  |
| *Lindera neesiana* (Wall. ex Nees) Kurz (Lauraceae) DLP212 | Siltimur (Np), Kutumb (Tam) | Sh: Wil | 1800-2700 | Common; Within district - Bajrabarahi | Fr | Raw | Oral | Diarrhea | [20, 35] |
| *Lippia nodiflora* (L.) Rich. (Verbinaceae) DLH113 | Bhringaraj, Kur Kure Jhar(NP), Kurkure (Tam) | Hb: Wil | 600-1400 | Common; Within district - everywhere | St/Wh pl | Powder/Paste | Topical | Alopecia, Headache ^NU^ |  |
| *Lobelia pyramidalis* Wall. (Lobeliaceae) DLH20 | Eklebir (Np), Eklebir (Tam) | Hb: Wil | 1100-2300 | Rare; Within district - daman | St/Lvs | Instillation/Juice/Paste | Topical | Eye infection, Bone aches ^NU^ |  |
| *Luffa cylindrica* (L.) Roem. (Cucurbitaceae) DLP201 | Ban Ghiraula (Np), Porol (Tam), Luffa (Eng) | Hb: Wil | 1200-1700 | Rare; Within district - Simbhanjyang | Fr | Powder | Oral | Jaundice ^NU^ |  |
| *Lycopodium clavatum* L. (Lycopodiaceae) DLP202 | Bhutle lahara (Np), Tamda (Tam), Club moss (Eng) | Sh: Wil | 1600-3600 | Common; Within district - Aghor | Wh pl | Paste | Topical | Rheumatism | [35] |
| *Lygodium japonicum* (Thunb.) Sw. (Lygodiaceae) DLP211 | Janai laharo (Np), Janai Lahara/Pinse (Tam) | Sh: Wil | 1000-3900 | Common; Within district - Hadikhola | Wh pl | Paste | Topical | Skin wounds | [35] |
| *Lyonia ovalifolia* (Wall.) Drude (Ericaceae) DLP218 | Angeri (Np), Domsing (Tam) | Tr: Wil | 1300-3300 | Common; Within district - Daman area, Aambhanjyang | St/Fr | Juice | Topical | Skin diseases, Scabies | [15, 36, 40, 43, 46, 50, 84] |
| *Mahonia napaulensis* DC. (Berberidaceae) DLP221 | Jamanemandro (Np), Bokipan (Tam) | Sh: Wil | 2000-2900 | Common; Within district - Daman | Br | Instillation | Topical | Eye itching | [15, 35] |
| *Mallotus philippensis* (Lam.) Mull. Arg. (Euphorbiaceae) DLP252 | Sindhure (Np), Pyongla (Tam), Kamala dye tree (Eng) | Tr: Wil | 150-1800 | Common; Within district - Hetauda area | Br | Powder | Oral | Curatortion/abortions, Stomach ache | [15, 47] |
| *Malus sylvestris* (L.) Mill. (Roseaceae) DLP329 | Syau (Np), Syau ( Tam), Apple (Eng) | Tr: Cul | 1200-3500 | Rare; Outside district - Rasuwa | Fr | Raw | Oral | To purify and increase blood ^NU^ |  |
| *Malvaviscus arboreus* Cav. (Malvaceae) DLP331 | Ghantiphool (Np), Ghanti (Tam) | Sh: Wil | 500-2500 | Common; Within district - Tistung | St/Lvs | Powder | Oral | Liver disorder ^NU^ |  |
| *Melia azedarach* L. (Meliaceae) DLP354 | Bakaino(Np), Chanyal (Tam), Bread tree( Eng) | Tr: Wil | 700-1100 | Common; Within district - Hetauda area | St/Sd | Paste/Powder | Topical/Oral | Headache, Fever | [15, 40] |
| *Morus serrata* Roxb. (Moraceae) DLP351 | Kimbu, kalo kafal(Np), Kalo kafal (Tam), Himalayan mulberry (Eng) | Tr: Wil | 1600-2400 | Common; Within district - Tistung, Palung, Aambhanjyang | St | Paste | Topical | Boils, Wounds ^NU^ |  |
| *Mucuna pruriens* (L.) DC. (Fabaceae) DLP308 | Kauso (Np), Kauso (Tam) | Hb: Wil | 150-1200 | Common; Within district - Bagmara | Sd | Paste | Topical | Diarrhea for children ^NU^ |  |
| *Musa paradisiaca* L. (Musaceae) DLP130 | Kera (Np), Moje (Tam), Banana(Eng) | Hb: Wil/Cul | <1800 | Common; Within district - everywhere | Fr | Raw | Oral | Diarrhea, Fruits edible |  |
| *Mussaenda frondosa* L. (Rubiaceae) DLH64 | Asarilahara (Np), Dong(Tam) | Sh: Wil | 1200 | Common; Within district - Aambhanjyang | Wh pl | Paste | Topical | Wounds in feet during summer | [15] |
| *Myrica esculenta* Buch.-Ham. ex D. Don (Myricaceae) DLP197 | Kaphal (Np), Naming (Tam) | Tr: Wil | 1200-2300 | Common; Within district - Tistung, Daman | Fr | Raw | Oral | Cholera, Fruits edible ^NU^ |  |
| *Nardostachys grandiflora* DC. (Valerianceae) DLP198 | Jatamansi (Np), Pange (Tam), Spikenard (Eng) | Hb: Wil | 3200-5000 | Rare; Outside district - Rasuwa | Rh | Powder | Oral | Tonic | [15] |
| *Nephrolepis cordifolia* (L.) Presl (Dryopteridaceae) DLP166 | Pani amala Np), Ambeli (Tam) | Hb: Wil | 500-2400 | Common; Within district - Hadikhola, Aambhanjyang, Tistung | Rh, Ysh | Paste | Topical | Fractures bones ^NU^ |  |
| *Nyctanthes arbor-tristis* L. (Oleaceae) DLH137 | Rudilo (Np), Khosora (Tam), Tree of sorrow (Eng) | Sh: Wil | 200-1200 | Common; Within district - everywhere | Wh pl | Decoction/hot infusion | Oral | Common cold | [15] |
| *Ocimum americanum* L. (Lamiaceae) DLH26 | Babari ( Np), Babari(Tam) | Hb: Wil | 1000 | Common; Within district - everywhere | Wh pl | Tea | Oral | Common cold, Fever | [15] |
| *Ocimum tenuiflorum* L. (Lamiaceae) DLH25 | Tulasi Np), Tulasi (Tam), French basil (Eng) | Hb: Cul | 400-900 | Common; Within district - everywhere | Wh pl | Tea | Oral | Common cold, Throat pain, Tonsilitis | [35] |
| *Oroxylum indicum* (L.) Kurz (Bignoniaceae) DLP16 | Tatelo( Np), Tare mendo (Tam), Broken bones (Eng) | Tr: Wil | 400-1400 | Common; Within district - aambhanjyang | Fr/Sd | Paste/Powder | Topical/Oral | Wounds, Dysentery | [15, 35] |
| *Osbeckia stellata* Buch.-Ham. ex D. Don (Melastomataceae) DLP27 | Gaure Phool, Rato Chulsi (Np), Gaure/aarbale(Tam) | Sh: Wil | 1300-2600 | Rare; Inside/Outside district - Tistung, Daman, Kafre, Sinndhupalchok | Wh pl | Paste | Topical | Arthritis ^NU^ |  |
| *Osmanthus suavis* King ex C. B. Clarke (Oleaceae) DLP345 | Silingi (Np), Silingi (Tam) | Sh: Wil | 2500-3800 | Common; Within district - Tistung | St/Br | Paste | Topical | Arthritis ^NU^ |  |
| *Osyris wightiana* Wall. ex Wight (Santalceae) DLP129 | Nundhiki (Np), Dhong (Tam) | Sh: Wil | 1100-2600 | Common; Within district - Hatiya | St | Paste | Topical | Arthritis ^NU^ |  |
| *Oxalis corniculata* L. (Oxalidaceae) DLP122 | Chari amilo (Np), Kunya dhap (Tam), Creeping sorrel (Eng) | Hb: Wil | 300-2900 | Common; Within district - everywhere | Wh pl | Instillation/Juice | Topical/Oral | Conjuctivitis, Migraine, Typhoid | [15, 35, 40] |
| *Paris polyphylla* Sm. (Liliaceae) DLP187 | Satuwa (Np), Kalchung(Tam), Love apple (Eng) | Hb: Wil | 1000-2900 | Rare; Within district - Simbhanjyang | Rh | Powder | Oral | Fever, Vomiting | [20] |
| *Persicaria chinensis* (L.) H. Gross (Polygonaceae) DLP143 | Kukur thotne (Np), Lahapangyu (Tam) | Hb: Wil | 1200-2900 | Common; Within district - Tistung | Ysh | Powder/Infusion | Oral | Astringent ^NU^ |  |
| *Phyllanthus emblica* L. (Euphorbiaceae) DLP13 | Amala (Np), Amble/tedu (Tam), Emblica myrobans (Eng) | Tr: Wil | 150-1400 | Common; Within district - everywhere | Fr | Powder | Oral | Cough, Gastritis | [35, 47] |
| *Pinus roxburghii* Sarg. (Pinaceae) DLP68 | Khote salla (Np), Salla (Tam), Chirpine (Eng) | Tr: Wil | 1100-2100 | Common; Within district - everywhere | Res | Raw/Tea | Oral | Cough, Chest ache ^NU^ |  |
| *Pinus wallichiana* A. B. Jacks. (Pinaceae) DLP61 | Gobre sallo (Np), Salla (Tam), Blue pine (Eng) | Tr: Wil | 1800-3300 | Common; Within district - Simbhanjyang | Res | Raw/Tea | Oral | Wounds, Worshipping | [7] |
| *Piper nigrum* L. (Piperaceae) DLP11 | Marich (Np), Marich (Tam), Long piper (Eng) | Hb: Wil | 100-1100 | Common; Within district - Hetauda area | Fr | Raw | Oral | Cough, Gastritis | [35] |
| *Plumbago zeylanica* L. (Plumbaginaceae) DLP10 | Batomuni Bato mathiko Jhar (Np), Pang (Tam) | Sh: Wil | 100-1300 | Common; Within district - Hadikhola | Wh pl | Powder | Oral | Retention of urine ^NU^ |  |
| *Potentilla fructicosa* L. (Rosaceae) DLP75 | Bajradanti (Np), Taptap (Tam), Bush cinquefoil (Eng) | Sh: Wil | 2000-5000 | Common; Within district - Daman, Simbhanjyang | Wh pl | Powder | Oral | Stomach problems | [35] |
| *Potentilla fulgens* Wall. ex Hook. (Rosaceae) DLP76 | Bajradanti (Np), Depse(Tam), Himalayan cinquefoil (Eng) | Hb: Wil | 1600-4800 | Rare; Outside district - Daman, Simbhanjyang | Wh pl | Powder | Oral | Tooth problems | [35] |
| *Prunus cornuta* (Wall. ex Royle) Steud. (Rosaceae) DLH26 | Baan aaru (Np), Ban aru (Tam), Wild peach (Eng) | Tr: Wil | 2100-3500 | Rare; Within district - Aghor, Simbhanjyang, Bagmara | Ysh, Br | Raw | Toothbrush | Poisoning ^NU^ |  |
| *Psidium guajava* L. (Myrtaceae) DLP81 | Amba (Np), Amba /belauti (Tam), Guava (Eng) | Tr: Wil | 450-1200 | Common; Within district - everywhere | Fr | Raw | Oral | Diarrhea, Fruits edible |  |
| *Pterocarpus marsupium* Roxb. (Fabaceae) DLP4 | Bijaya saal (Np), Bijaya saal (Tam) | Tr: Wil | 100-1000 | Rare; Outside district - Mahendranagar district | St/Wd | Powder/Infusion | Oral | Cooling of body during hot seasons ^NU^ |  |
| *Pycnoporus cinnabarinus* (Jacq. ) Fr. (Polyporaceae) DLP53 | Rato chau (Np), Chau (Tam) | : Wil | 1000-2500 | Rare; Outside district - Rasuwa | Wh pl | Instillation | Topical | Ear infection , Controls to decrease pus formation ^NU^ |  |
| *Quercus lanata* Sm. (Fabaceae) DLP401 | Banjha (Np), Pepker (Tam) | Tr: Wil | 460-2600 | Common; Within district - Daman, Simbhanjyang | Wd/Res | Paste | Topical | Scorpion sting |  |
| *Raphanus sativus* L. (Brassicaceae) DLP7 | Mula (Np), Labu (Tam), Radish (Eng) | Hb: Wil | 500-3500 | Common; Within district - everywhere | Rt, Lvs | Juice/Paste | Topical | Cuts, To releive hotness |  |
| *Reinwardtia indica* Dumort. (Linaceae) DLP342 | Pyauli (Np), Nagachhe (Tam), Winter flax (Eng) | Hb: Wil | 300-2300 | Common; Within district - everywhere | Lvs | Paste | Topical | Boils, Bruises | [35] |
| *Rhododendron arboreum* Sm. (Ericaceae) DLH108 | Laligurans (Np), Paramhendo/pat (Tam), Rhododendron (Eng) | Tr: Wil | 1500-3300 | Common; Within district - Daman, Simbhanjyang | Fl | Raw | Oral | Throat obstruction especially fish bones | [35, 38] |
| *Rubus ellipticus* Sm. (Roseaceae) DLH104 | Aiselu (Np), Pulung(Tam), False black berry (Eng) | Sh: Wil | 1700-2300 | Common; Within district - everywhere | Rt/Br/Fr | Juice/Raw | Topical/Oral | Cuts, fuirts are edible | [15, 36, 84] |
| *Sambucus hookeri* Rehder (Caprifoliaceae) DLP20 | Galeni (Np), Galeni(Tam) | Sh: Wil | 1400-2400 | Common; Within district - Tistung | St/Fr/Sd | Paste | Topical | Snake bite ^NU^ |  |
| *Schima wallichii* (DC.) Korth. (Theaceae) DLP6 | Chilaune (Np), Kyasim(Tam), Needle wood (Eng) | Tr: Wil | 900-2100 | Common; Within district - Aambhanjyang | Br/Lvs | Paste | Topical | Wounds | [35] |
| *Schoenoplectus juncoides* (Roxb.) Palla (Cyperaceae) DLH32 | Gudmothe (Np), Gudmothe (Tam) | Hb: Wil | 1500-2700 | Common; Within district - Raksirang, Sarikhet | Wh pl | Paste | Topical | Typhoid ^NU^ |  |
| *Scutellaria repens* Buch.-Ham. ex D. Don (Lamiaceae) DLP57 | Seto lahara (Np), Kudu mran (Tam) | Hb: Wil | 600-2100 | Common; Within district - Aambhanjyang | Ysh/Wh pl | Paste, Juice | Topical, oral | Fractured bones, Fever | [35, 45, 47] |
| *Sesamum orientale* L. (Pedaliaceae) DLP17 | Kalo til (Np), Grangasa (Tam), Benne (Eng) | Hb: Wil | 600-2400 | Common; Within district - Hatiya | Sd | Paste | Topical | Leucoderma , Skin diseases | [15, 68, 85, 86] |
| *Shorea robusta* Gaertn. (Dipterocarpaceae) DLP2 | Saal (Np), Agrakh (Tam), Saal tree (Eng) | Tr: Wil | 150-1500 | Common; Within district - Hadikhola | Res | Paste | Topical | Joints aches | [15, 36, 68, 86] |
| *Sida cordifolia* L. (Malvaceae) DLP5 | Balu (Np), Mesi (Tam) | Hb: Wil | 500-1100 | Common; Within district - Aambhanjyang | Wh pl | Instillation | Topical | Eye problems and whitening of cornea ^NU^ |  |
| *Smilax zeylanica* L. (Smilacaceae) DLP333 | Kukurdaino (Np), Chatalang (Tam) | Sh: Wil | 150-1500 | Common; Within district - Hadikhola | Lvs | Raw | Toothbrush | Toothaches ^NU^ |  |
| *Solanum nigrum* L. (Solanaceae) DLP329 | Kaligadhi (Np), Chema (Tam) | Hb: Wil | 900-2900 | Common; Within district - everywhere | Lvs | Raw | Oral | Malnutrition in children ^NU^ |  |
| *Solanum surattense* Burm. f. (Solanaceae) DLP157 | Kanthakari (Np, Tam), Indian salmon (Eng) | Sh: Wil | 300-900 | Common; Within district - everywhere | Fr | Paste | Topical | Headache ^NU^ |  |
| *Strychnos nux-vomica* L. (Loganiaceae) DLP143 | Patuwa (Np), Patuwa (Tam) | Tr: Wil | <1300 | Common; Within district - Hatiya | Fr | Juice/Infusion/Powder | Oral | Anti-rabies ^NU^ |  |
| *Swertia chirayita* (Roxb. ex Fleming) Karsten (Gentiniaceae) DLH22 | Chiraito (Np), Timda (Tam), Chiretta(Eng) | Hb: Wil | 1500-2500 | Common; Within district - Daman, Aghor | Wh pl | Powder | Oral | Fever | [35, 46] |
| *Syzygium cumini* (L.) Skeels (Myrtaceae) DLP15 | Kyamuno (Np), Jabu (Tam), Black berry (Eng) | Tr: Wil | 300-1200 | Common; Within district - Hetauda area | Br/Fr/Sd | Powder | Oral | Typhoid ^NU^ |  |
| *Taraxacum officinale* Wigg. (Asteraceae) DLP36 | Tuki phool (Np), Hyo mran (Tam) | Hb: Wil | 1000-4000 | Common; Within district - Aambhanjyang | Lvs | Infusion | Oral | Bodyache ^NU^ |  |
| *Taxus wallichiana* Zucc. (Taxaceae) DLP33 | Lautha salla (Np), Siding (Tam), Taxus (Eng) | Tr: Wil | 2200-3400 | Rare; Within district - Daman | Lvs | Cooked, raw | Oral | Consumed as diet, Respiratory problems, For worshipping | [15, 20, 35, 68, 86] |
| *Tectaria macrodonta* (Fee) C.Chr. (Aspidiaceae) DLP3011 | Niuro (Np), Toplign degni (Tam) | Hb: Wil | <1100 | Common; Within district - everywhere | Lvs | Raw/Powder | Oral | Stomach ache ^NU^ |  |
| *Terminalia bellirica* (Gaertn.) Roxb. (Combreaceae) DLP308 | Barro (Np), Barro (Tam), belleric myrobolans (Eng) | Tr: Wil | 300-1100 | Common; Within district - Hetauda area | Fr | Raw/Powder | Oral | Cough, Gastritis | [38, 47] |
| *Terminalia chebula* Retz. (Combretaceae) DLP296 | Harro (Np), Harro(Tam), chebulic myrobolans (Eng) | Tr: Wil | 150-1100 | Common; Within district - Hetauda area | Fr | Powder | Oral | Cough, Gastritis | [15, 35, 38, 47, 68, 84, 85] |
| *Thalictrum foliolosum* DC. (Ranunculaceae) DLP257 | Dampate (Np), Bathuri (Tam) | Hb: Wil | 1300-3400 | Common; Within district - Bagmara area | Wh pl | Powder | Oral | Asthma, Fever | [35] |
| *Thysanolaena maxima* (Roxb.) Kuntze (Poaceae) DLP107 | Amriso (Np), Karuti chhe/phus (Tam) | Hb: Wil | 100-2000 | Common; Within district - everywhere | Rt | Powder, soup | Oral | Malarial fever ^NU^ |  |
| *Trachyspermum ammi* (L.) Sprague (Apiaceae) DLP93 | Jwano (Np), Jwano (Tam) | Hb: Wil | <1000 | Common; Within district - Raksirang, Sarikhet | Ysh | Infusion | Oral | Cough ^NU^ |  |
| *Trichilia connaroides* (Wight & Arn.) Bentv. (Meliaceae) DLP65 | Aankhtaruwa (Np), Bankhataruwa (Tam) | Tr: Wil | 700-2400 | Common; Within district - Aambhanjyang | St/Fr/Sd | Juice/Paste | Topical | Surface itching in skin | [15, 35, 36, 84] |
| *Trichosanthes anguina* L. (Cucurbitaceae) DLP94 | Chichindo (Np), Chichinda (Tam), Snake guard(Eng) | Hb: Wil | 1000-1800 | Common; Within district - everywhere | Sd | Paste | Topical | Snake bite ^NU^ |  |
| *Trigonella foenum-graceum* L. (Fabaceae) DLH14 | Methi (Np), Methi (Tam), bird’s foot (Eng) | Hb: Wil | 100-1500 | Common; Within district - everywhere | Sd | Powder | Oral | Cough | [15, 84, 85] |
| *Triticum aestivum* L. (Poaceae) DLP217 | Gaun (Np), Kwa (Tam), Wheat (Eng) | Hb: Cul | 1200-3000 | Common; Within district - everywhere | Sd | Paste | Oral | Wounds | [15, 86] |
| *Urtica dioica* L. (Urticaceae) DLH69 | Sishnu (Np), Polo (Tam), Stinging nettle (Eng) | Hb: Wil | 3000-4500 | Common; Within district - everywhere | Rt/Lvs | Paste | Oral | Bone fractures | [47] |
| *Valeriana jatamansii* Jones (Valerianceae) DLH96 | Sugandhawala (Np), Brasen (Tam), Valerian (Eng) | Hb: Wil | 1500-3300 | Rare; Outside district - Rasuwa, Simbhaynjayang | Rh/Wh pl | Paste | Topical | Fire burns | [7] |
| *Vitex negundo* L. (Verbenaceae) DLH74 | Simali (Np), Binyal (Tam), Chinese chaste tree (Eng) | Tr: Wil | <1600 | Common; Within district - everywhere | Ysh/Lvs | Powder/Fuming | Nasal | Sinusitis | [35] |
| *Vitis vinifera* L. (Vitaceae) DLP48 | Angur (Np), Angur (Tam), Grapes (Eng) | Sh: Wil | 100-1000 | Rare; Outside district - Bara, Parsa | Ysh | Powder/Juice | Oral | Fever ^NU^ |  |
| *Woodfordia fruticosa* (L.) Kurz (Lythraceae) DLP236 | Dhayero (Np), Jamjasa/syakate (Tam), Fire-flame bush (Eng) | Sh: Wil | 200-1800 | Common; Within district - everywhere | Lvs/Fl | Raw | Oral | Dysentery, Diarrhoea | [15, 35, 36, 38, 46, 47, 68, 84, 85] |
| *Zizyphus mauritiana* Lam. (Rhamnaaceae) DLP150 | Bayer (Np), Bayer (Tam), Indian plum , Chinese date (Eng) | Tr: Wil | 200-1200 | Common; Within district - Hetauda area | Rt/Fr | Raw | Oral | Headache, Fever | [35] |

**Notes**

^A^ Np, Nepali names; Tam, Tamang names; Eng, English names.

^B^ Hb, herbs; Sh, shrubs; Tr, Tree; Wil, Wild grown plant; Cul, Cultivated plant.

^C^B, bulbs; Br, barks; F, fruits; Fl, flowers; L, leaves; R, roots; Re, resin; Rh, rhizome; Sd, seeds; Sh, shoot; St, stem; T, tubers; Wd, wood; Wp, whole plant; Ysh, Young shoots.

^D^NU, New reported uses that were not mentioned in compared studies.
