# Supplementary material for: Medicinal plants used by the Tamang community in the Makawanpur district of central Nepal
Source: J Ethnobiol Ethnomed. 2014 Jan 10;10:5. doi: 10.1186/1746-4269-10-5 (PMC3904474; doi:10.1186/1746-4269-10-5)
Supplement: Additional file 3: Table S3 — Comparison of local use and phytochemical and pharmacological studies of medicinal plants [94-186]. [file 1746-4269-10-5-S3.docx]

**Additional file 3: Table S3** Comparison of local use and phytochemical and pharmacological studies of medicinal plants [95-187].

| **Plant names** | **Indigenous uses** | **Pharmacological/phytochemical studies (Literature review)** | **Indigenous use coherent with known pharmacological/phytochemical studies** |
| --- | --- | --- | --- |
| *Acacia catechu* (L. f.) Willd. | Joints aches, Fever | Ethyl acetate extract showed significant antipyretic activity in albino rats [95], Taxifolin isolated from the plant is anti-inflammatroy in action [96], Ethanolic extracts are found to possess antimicrobial activity [97]. Bark extract was tested against different bacteria (*E. coli*, *Shigella boydii*, *Stahylococcus aureus* and *Pseudomonas aeruginosa*) and there was positive effect on P. aeruginosa [98]. Ethanolic extract of plant was tested against *Enterococcus faecalis*, *E. coli*, *S. aureus*, *Proteus* *mirabilis*, *P*. *aeruginosa*, *Salmonella* *typhi*, *Shigella* *dysenteriae* and *Staphylococcus* *aureus* and had posetive effect on all bacteria acteria [99]. | Yes |
| *Acacia pennata* (L.) Willd. | Wounds | Analgesic activity was evaluated using chemical (acetic acid and formalin) and mechanical stimuli in albino mice. It was proved that *A. pennata* has properties of analgesic or anti-inflammatory [100]. | Yes |
| *Achyranthes bidentata* Blume | Stomatitis, Common cold | Triterpenoid saponins isolated from the plant are useful in treating canker sores, toothache, bleeding nose bleeds [101]. | Yes |
| *Aconitum ferox* Wall. ex Ser. | Toothaches | Alkaloid extracts possibly are as anti-inflammatory properties [102]. | Yes |
| *Acorus calamus* L. | Cough, Fever | The methanolic or petroleum ether extract of *A. calamus* rhizome is proved to be antimicrobial in nature [103–105]. Rhizome extract pr essential oil of the plant showed antimicrobial effect on different kinds of bacteria such as *B. subtilis*, *E. coli*, *Klebsiella. pneumniae*, *K. pneumoniae*, *P. aeruginosa*, *P. mirabilis*, *S. aureus*, *S. boydii* and *S*. *dysenteriae* [99, 106–110]. | Yes |
| *Aegle marmelos* (L.) Correa | Dysentery, Diarrhoea | Methanolic extract and aqueous extracts are effective against dysentery causing *Salmonella typhi* [111], diarrhoea causing *E. coli* [112], *Giardia* and rotavirus [113]. Fruits and leaf extract of plant showed inhibitory effect on growth of *E. coli*, *K*. *pneumniae*, *P*. *aeruginosa*, *S. aureus* and *S. dysenteriae* [98, 106, 109]. | Yes |
| *Aloe vera* (L.) Burm. f. | Fire burns | Chromones, glycoprotein, Hermones help in wound healing and have anti-inflammatory in nature [114, 115]. | Yes |
| *Alstonia scholaris* (L.) R. Br. | Abortions | Oral administration (100 mg/kg/day per animal for 21 days) showed significant luteolytic and anti-implantational effect in male albino rats [116]. *B*ark extract showed a significant antifertility effect in male rats at the dose rate of 200 mg/day for 60 days [117]. Lupeol acetate extracted from the plant showed male fertility reduced by 100 % (10 mg/rat/day) [118]. | Yes |
| *Amaranthus spinosus* L. | Skin diseases | It contains several falvonoids and volatile oils that are antimicrobial and antiseptic in nature [119]. Methanolic extract of plant or rhizome extract showed antimicrobial effect against *Bacillus. subtilis*, *Candida. albicans*, *E. coli*, *S. paratyphi*, , *S. aureus* [109, 120]. | Yes |
| *Ananas comosus* (L.) Merr. | Fever | Several vitamins present in the plant are useful in curing various diseases [121]. | Partial |
| *Artemisia indica* Willd. | Scabies | Essential oil from the aerial parts of was found to be antimicrobial in nature [122]. Leaves extract of plant showed antimicrobial effect gainst *B. subtilus*, *S*. a*ureus*, *E*. *coli* and *P*. *aeruginosa* [123]. | Yes |
| *Artocarpus heterophyllus* Lam. | Boils, Bruises | artocarpain-H, flavanoids, cycloheterohyllin, artonins B, and artocarpanon, artocarpanone extracted from fruits and stem show anti-inflammatory behavior [124]. | Yes |
| *Asparagus racemosus* Willd. | Better lactation | Aqueous extract of roots increased the weight of mammary glands in post-partum and oestrogen-primed rats [125]. Alcoholic extract of rhizome in adult pregnant female albino rats had an oestrogenic effect on the female mammary glands and genital organs [126]. It has also been observed an in increase in milk secretion after administration of the plant [127]. | Yes |
| *Astilbe rivularis* Buch.-Ham. ex D. Don | Energizer for delivered women | β-amyrin, β-sitosterol, β-peltoboykinolic acid, astilbic acid and quercetin [128]. | Unknown |
| *Azadirachta indica* A. Juss. | Scabies, Skin diseases | Nimbidin present in plant is anti-inflammatory in nature [129]. | Partial |
| *Bauhinia variegata* L. | Dysentery | Alcoholic extract of the plant is found to be antimicrobial in activity [130]. | Yes |
| *Berberis aristata* DC. | Eye infection | The use of plant extract in conjunctivitis widespread [131]. Bark extract showed antimicrobial activity against *B*. *subtilis*, *E*. *coli*, *Macrococcus* sp., *P*. *aeruginosa*, *P*. *aeruginosa*, *S*. *aureus*, *S*. *boydii* and *S*. *typhii* [108, 132–135]. | Yes |
| *Bergenia ciliata* (Haw.) Sternb. | Energizer for delivered women | The different kinds of amino acids and mineral elements possess good nutritive value [136] and considered as medicinal [137]. | Yes |
| *Cannabis sativa* L. | Diarrhea | Cannabinol, Cannabinoids and several vitamins B1, B2, etc. are useful for gastro-intestinal tract as they stimulate intestinal mucosa causing an increase in secretions and peristalsis [138, 139]. Fruit extract of plant was tested against *E*. c*o*li, *S*. *boydii*, *S*. *aureus*, and *P*. *aeruginosa* and had positive effect on *S*. *boydii*, *S*. *aureus* and *P*. *aeruginosa* [98]. | Yes |
| *Centella asiatica* (L.) Urb. | Purification and retention of urine, Headache | Secondary metabolites such as asiaticoside, madecassoside, brahmoside, brahmissoside are useful in lowering blood pressure and also as diuretic [138]. | Partial |
| *Citrus aurantifolia* (Christ.) Swingle | Wounds in eyes | Limonene, beta-pinene, gamma-terpinene , citral [140], Isosinensetin, Xanthyletin [141]. | Unknown |
| *Cucumis sativus* L. | Snake bite | cucurbitacins, cucumegastigmanes I and II, cucumerin A and B, vitexin, orientin, isoscoparin 2″-O-(6‴-(E)-p-coumaroyl) glucoside, apigenin 7-O-(6″-O-p-coumaroylglucoside), etc. [142]. | Unknown |
| *Cynodon dactylon* (L.) Pers. | Skin wounds | Glycerin, Thymol, Conhydrin, Linoleic acid ethyl ester and other complex compounds are useful as antimicrobial, antiseptic, anticancer, etc. [143]. It is also proved as anti-inflammatory in nature when tested in albino rats [144]. Whole plant extract was tested against *B*. *subtilus*, *S*. au*r*eus, *E*. *coli*, *Macrococcus* sp, *P*. *aeruginosa* and *S*. *typhii*. There was positive effect on all bacteria type [98, 135]. | Yes |
| *Desmodium multiflorum* DC. | Typhoid | Flavonoids, desmodianones A, desmodianones B and desmodianones show antimicrobial activity [145]. | Yes |
| *Entada phaseoloides* (L.) Merr. | Constipation, Iron deficiency | Saponins present in the plant are useful in indigestion [101]. | Partial |
| *Eupatorium adenophorum* Spreng. | To control bleeding during cuts in skin | 4'-methyl quercetagetin 7-O-(6”-O-E-caffeoylglucopyranoside), caffeic acid, eupalitin, eupalitin 3-O-β-D-galactopyranoside, quercetagetin 7-O-(6”-O-acetyl-β-D-glucopyranoside) [146], Eupatoranolide [141]. | Unknown |
| *Euphorbia hirta* L. | Cuts, Snake bite | Methanol extract of the plant showed antimicrobial activity [147, 148]. Aqueous extract of plant showed anti-inflammatory [149, 150]. | Yes |
| *Ficus religiosa* L. | Ear infection and pain | Anti-inflammatory and analgesic property of the plant was proved by different researchers [151–153]. | Yes |
| *Glycyrrhiza glabra* L. | Gastritis | Different chemicals (glycorrhizin, mucilage, flavonoids, glycyrrhetinic acid, saponin, glabridin, tannic acid, 2-β-glucuronosyl, glucuronic acid) present in the plant are useful for intestinal disorders [154]. | Yes |
| *Jatropha curcas* L. | Wounds in the feet during summer due to mud | Ethanolic, methanolic and water extracts of the stem bark of the plant were found to be antimicrobial in nature [155]. Leaves extract of plant was tested against *B*. *subtilus*, *S*. *aureus*, *E*. *coli* and *P.* *aeruginosa* and had positive effect *S*. *aureus* [123]. | Yes |
| *Juglans regia* L. | Wounds, Tooth ache | Ethanolic extracts of leaves exhibited potent anti-inflammatory activity [156]. Bark contains juglone, betulinic acid, regiolone and β-sitosterol [157]. Juglone contains a naphthaquinone and naphthoquinones and they have antibacterial, antifungal, antiviral, insecticidal and anti-inflammatory properties [158]. | Yes |
| *Justicia adhatoda* L. | Cough, Fever | Vasicine present in the plant is widely effective in wide ranges of diseases [159]. Leaves extract of plant showed inhibitory effect against the growth of *P*. *aeruginosa*, *S*. *aureus* and *B*. *subtilis* bacteria types [134]. | Partial |
| *Lawsonia inermis* L. | To control hairfall and dendruf with small pinpleson head | Alpha-ionone, beta-ionone, gallic acid, lawsone are bactric bactericidal and fungicidal [160]. | Yes |
| *Lindera neesiana* (Wall. ex Nees) Kurz | Diarrhea | Oil extracted from fruits are antimicrobial in nature [161]. | Partial |
| *Lycopodium* *clavatum* L. | Rheumatism | Chloroform extract and the alkaloid fraction displayed marked anti-inflammatory effect when tested in mice [162]. | Yes |
| *Lyonia ovalifolia* (Wall.) Drude | Skin diseases, Scabies | Andromedotoxin and lyoniols present in the plants are highly toxic in nature [163] and antimicrobial [164]. Methanolic extract of plant or leaves or apical buds showed antimicrobial effect against *B*. *subtilis*, *C*. *albicans*, *E*. *coli* *Macrococcus* sp., *P*. *aeruginosa*, *S*. *boydii*, *S*. *aureus*, and *S*. *typhii* [98, 108, 120]. | Yes |
| *Mallotus philippensis* (Lam.) Mull. Arg. | Curatortion/abortions, Stomach ache | The administration of seed extracts to mice reduced serum levels of gonadotropins probably by affecting hypothalamic/pituitary axis and also levels of FSH and LH might have affected steroidogenesis in the ovary which probably contribute in poor quality of eggs, reduced number of ovulated eggs and corpora lutea which might have affected establishment and maintenance of pregnancy [165]. Methanolic extract of plant showed inhibitory effect on growth of *S*. *dysenteriae* which causes dysentery in human [98, 120, 134]. | Partial |
| *Musa paradisiaca* L. | Diarrhea, Fruits edible | Serotonin, norepinephrine present in the fruit are helpful in stimulating the smooth muscle of the intestine and help in digestion [163], flavonoid, leucocyanidin protects the gastric mucosa when tested in apirin-induced mice [166], queous extract of unripe fruit peels and leaves of the plant are antimicrobial in nature [167]. | Yes |
| *Myrica esculenta* Buch.-Ham. ex D. Don | Cholera, Fruits edible | Bark, fruits and Stem extracts of the plant showed antimicrobial effect on *B*. *subtilis*, *Macrococcus* sp, *P*. *aeruginosa*, *P*. *aeruginosa*, *S*. *aureus*, *S*. *aureus*, *S*. *boydii* [134, 135]. | Yes |
| *Nardostachys grandiflora* DC. | Tonic | Ethanol extract of roots are anticonvulsant in activity and are a nervous system stimulant [168] and is useful for increasing memory [169]. | Yes |
| *Nyctanthes arbor-tristis* L. | Common cold | Different extracts of the plant is found to be antimicrobial in nature [170]. | Partial |
| *Oxalis corniculata* L. | Conjuctivitis, Migraine, Typhoid | A methanol and ethanol extracts of leaf of the plant is found to be antibacterial in nature [171]. | Partial |
| *Paris polyphylla* Sm. | Fever, Vomiting | A methanolic extract is gastro protective in nature [172]. Leaves or rhizome extracts were tested against *B*. *subtilus*, *E*. *coli*, *P*. *aeruginosa*, *S*. *aureus*, *S*. *aureus* and *S*. *boydii* and had positive effect only on *E*. *coli* and *S*. *boydii* [108, 123]. | Partial |
| *Phyllanthus emblica* L. | Cough, Gastritis | The volatile components were found to be antimicrobial in nature [173]. Ethanolic extract of plant or fruit extract was tested against *E*. *coli*, *E*. *faecalis*, *P*. *aeruginosa*, *P*. *irabilis*, *S*. *typhi*, *S*. *aureus*, *S*. boydii and S. dysenteriae and had positive effect on *E*. *faecalis*, P. *aeruginosa*, *P*. *mirabilis* and *S*. *aureus* [99, 108]. | Yes |
| *Pinus wallichiana* A. B. Jacks. | Wounds, Worshipping | It has rosin contains abietic acid and turpentine. Turpentine contains α-pinene, β-pinene undecane, dodecane, tridecane and sesquiterpenes, isopimaric acid and lambertianic acid [128]. | Unknown |
| *Piper nigrum* L. | Cough, Gastritis | Piperine, volatile oil, protein, l-phyllandrene, caryophyllene have stimulant effect on digestive system [174]. | Partial |
| *Potentilla fulgens* Wall. ex Hook. | Tooth problems | It is antibacterial and anti-inflammatory in nature [175]. | Yes |
| *Psidium guajava* L. | Diarrhea, Fruits edible | The methanol extract of the leaves exhibited antispasmodic and anti-diarrhoeal effects by inhibiting intestinal motility and preventing castor oil-induced diarrhoea [176]. Leaves extract of plant showed antimicrobial effect on *S*. *aureus* and *P*. *aeruginosa* [106]. Quercetin present in the fruit showed significant anti-diarrhoeal activity [177]. Alactose-specific lectin isolated from fruit ripe prevent *E. coli* from binding on the intestinal wall and and preventing infection that causes diarrhoea [178]. | Yes |
| *Raphanus sativus* L. | Cuts, To releive hotness | Raphanin present in the plant is bactericidal in nature [174]. | Partial |
| *Rhododendron arboreum* Sm. | Throat obstruction especially fish bones | Betulinic acid [141]. | Unknown |
| *Scutellaria repens* Buch.-Ham. ex D. Don | Fractured bones, Fever | Flavonoids and phenylethanoids [179]. | Unknown |
| *Swertia chirayita* (Roxb. ex Fleming) Karsten | Fever | The aqueous extract of root was found to be antipyretic comparable (150 mg/kg body weight, p.o.) [180]. | Yes |
| *Syzygium cumini* (L.) Skeels | Typhoid | Fruit extract of plant exhibited antimicrobial effect against stomach disoder causing bacteria types such as *B*. *subtilis*, *E*. *faecalis*, *P*. *aeruginosa*, *P*. *aeruginosa*, *P*. *mirabilis*, *S*. *aureus* and *S*. *typhi* [99, 106, 107]. | Yes |
| *Taraxacum officinale* Wigg. | Bodyache | Ethanolic extract of the plant showed reduction in writhing response to phenylquinone in [181]. | Yes |
| *Taxus wallichiana* Zucc. | Consumed as diet, Respiratory problems, For worshipping | The plant is antimicrobial in nature [182] and possibly help to overcome respiratory problem. | Yes |
| *Terminalia bellirica* (Gaertn.) Roxb. | Cough, Gastritis | Fruit extract of plant exhibited antimicrobial effect against stomach disoder causing bacteria types such as *B*. *subtilis*, *E*. *faecalis*, *P*. *aeruginosa*, *P*. *aeruginosa*, *P*. *mirabilis*, *S*. *aureus* and *S*. *typhi* [99, 106, 107]. | Yes |
| *Terminalia chebula* Retz. | Cough, Gastritis | Fruit extract or ethanolic extract of plant showed positive effect on stomach disorder causing *B*. *subtilis*, *K*. *pneumniae*, *P*. *aeruginosa*, *S*. *aureus* and *S*. *boydii* [106–109, 183]. | Yes |
| *Valeriana jatamansii* Jones | Fire burns | Seventy-two compounds have been idenditifed and some of the major amounts are isovaleric acid, patchouli, 3-methyl pentanoic acid, 1-ethyl-4,4-dimethyl-cyclohex-2-en-1-ol, neocembreneA, 3-methylvaleric acid, maaliol, Iridoids, and valeriananoids A, B and C [128]. | Unknown |
| *Vitex negundo* L. | Sinusitis | The plant possess antimicrobial activity [184]. The study carried out by orally treating a water extract of the leaves to rats show anti-inflammatory or analgesic effects [185]. | Yes |
| *Woodfordia fruticosa* (L.) Kurz | Dysentery, Diarrhoea | The essential oil of leaves are sesquiterpenoids (β-caryophyllene, γ-curcumene, germacrene-D, β-selinene, elemol) and monoterpenoids (α-pinene, 2,6 dimethyl 1,3,5,7 octatetraene) have shown antimicrobial activity against diarrhea and dysentery causing bacteria [186]. Methanol, chloroform and hexane extracts of leaves and flowers exhibited the antimicrobial activity against diarrhea and dysentery causing bacteria [187]. Methanol, chloroform and hexane extracts of leaves and flowers exhibited the antimicrobial activity against diarrhea and dysentery causing bacteria [171]. Methanolic extract of plant or flower extract showed inhibitory effect on *B*. *subtilis*, *C*. *albicans*, *E*. *coli*, *K*. *pneumoniae*, *P*. *aeruginosa*, *S*. *paratyphi*, *S*. *typhi*, *S*. *dysenteriae*, *S*. au*r*eus and *Vibrio cholerae* [109, 120]. | Yes |
